# PC2P: parameter-free network-based prediction of protein complexes

**DOI:** 10.1093/bioinformatics/btaa1089

**Published:** 2021-01-08

**Authors:** Sara Omranian, Angela Angeleska, Zoran Nikoloski

**Affiliations:** Bioinformatics, Institute of Biochemistry and Biology, University of Potsdam, 14476 Potsdam, Germany; Systems Biology and Mathematical Modeling, Max Planck Institute of Molecular Plant Physiology, 14476 Potsdam, Germany; Mathematics Department, University of Tampa, Tampa, FL 33606, USA; Bioinformatics, Institute of Biochemistry and Biology, University of Potsdam, 14476 Potsdam, Germany; Systems Biology and Mathematical Modeling, Max Planck Institute of Molecular Plant Physiology, 14476 Potsdam, Germany; Bioinformatic Department, Centre of Plant Systems Biology and Biotechnology (CPSBB), 4000 Plovdiv, Bulgaria

## Abstract

**Motivation:**

Prediction of protein complexes from protein–protein interaction (PPI) networks is an important problem in systems biology, as they control different cellular functions. The existing solutions employ algorithms for network community detection that identify dense subgraphs in PPI networks. However, gold standards in yeast and human indicate that protein complexes can also induce sparse subgraphs, introducing further challenges in protein complex prediction.

**Results:**

To address this issue, we formalize protein complexes as biclique spanned subgraphs, which include both sparse and dense subgraphs. We then cast the problem of protein complex prediction as a network partitioning into biclique spanned subgraphs with removal of minimum number of edges, called coherent partition. Since finding a coherent partition is a computationally intractable problem, we devise a parameter-free greedy approximation algorithm, termed Protein Complexes from Coherent Partition (PC2P), based on key properties of biclique spanned subgraphs. Through comparison with nine contenders, we demonstrate that PC2P: (i) successfully identifies modular structure in networks, as a prerequisite for protein complex prediction, (ii) outperforms the existing solutions with respect to a composite score of five performance measures on 75% and 100% of the analyzed PPI networks and gold standards in yeast and human, respectively, and (iii,iv) does not compromise GO semantic similarity and enrichment score of the predicted protein complexes. Therefore, our study demonstrates that clustering of networks in terms of biclique spanned subgraphs is a promising framework for detection of complexes in PPI networks.

**Availability and implementation:**

https://github.com/SaraOmranian/PC2P.

**Supplementary information:**

Supplementary data are available at *Bioinformatics* online.

## 1 Introduction

Identification of protein complexes can provide mechanistic insights into principles of cellular organization and functions. Protein complex formation can affect different cellular processes, from signaling cascades (Pawson and Nash, 2000) to metabolism (Sweetlove and Fernie, 2018) via alteration of enzyme abundance, enzyme activity and substrate specificity (Reyes-Turcu *et al.*, 2009). Due to technological developments, protein–protein interactions (PPIs) underlying protein complexes can now be profiled by the combination of yeast two hybrid (Fields and Sternglanz, 1994), co-immunoprecipitation (Lin and Lai, 2017), affinity purification-mass spectrometry (Bauer and Kuster, 2003), split luciferase complementation assay (Fujikawa and Kato, 2007) and correlation of elution profiles from size exclusion chromatography (McBride *et al.*, 2019). The resulting interactions comprise large-scale PPI networks which are available for well-studied model organisms, from *Escherichia coli* (Rajagopala *et al.*, 2014) and *Saccharomyces cerevisiae* (yeast) (Collins *et al.*, 2007; Gavin *et al.*, 2006; Krogan *et al.*, 2006; Stark, 2006) to *Homo sapiens* (human) (McDowall *et al.*, 2009; Szklarczyk *et al.*, 2015).

The increase in size and quality of PPI networks is paralleled by the development of algorithms for mining of these networks. In particular, there has been a considerable interest in design of algorithms for prediction of protein complexes based on PPI networks (Wu *et al.*, 2020; Zahiri *et al.*, 2020). Unbiased comparison of these algorithms has been facilitated by the generation of gold standards of protein complexes for different model organisms, such as: MIPS, SGD and CYC2008 for yeast (Hong *et al.*, 2007; Mewes, 2004; Pu *et al.*, 2009) and CORUM for human (Giurgiu *et al.*, 2019). The approaches for prediction of protein complexes based on PPI networks can be divided into supervised and unsupervised. For instance, one supervised approach determines the probability that a subnetwork of interactions is a part of a given complex by training a neural network with structural features of the subnetwork (Shi *et al.*, 2011). However, due to the relatively small number of complexes documented in different gold standards and their diverse structural properties, the increase in performance is marginal in comparison to the unsupervised approaches.

The unsupervised approaches for protein complex prediction based on PPI networks rely on detecting network clusters based on different network properties and concepts (Zahiri *et al.*, 2020). A unifying idea of unsupervised approaches is that protein complexes are embedded in dense subnetworks. Such dense subnetworks are identified either by merging and growing clusters or by network partitioning, following different criteria. The merging and growing of clusters usually starts with small subnetworks, e.g. cliques, which are expanded based on different similarity measures, such as the size of overlaps. These network clusters are further combined with additional biological knowledge, from ontologies or evolutionary relationships, to increase the prediction accuracy. Therefore, the unsupervised approaches have been categorized into those which rely only on network clustering and those which consider additional biological information (Srihari and Leong, 2013).

**Fig. 1.**
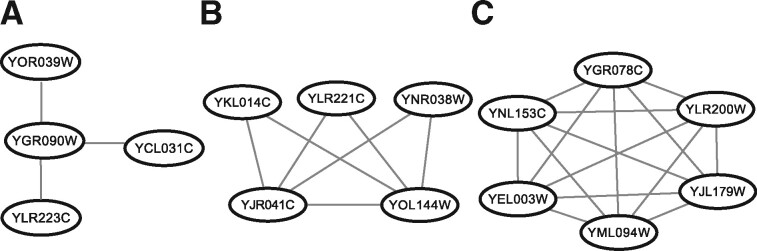
Protein complexes vary in density. Shown are three proteins complexes in yeast (**A**) CURI, inducing a star, (**B**) Npa2p-containing subcomplex, inducing a biclique spanned subgraph and (**C**) Prefoldin, forming a clique

Comparative analyses of the performance of the existing unsupervised approaches for protein complex prediction demonstrate that they exhibit small recall (at most ∼65%), largely due to the assumption that protein complexes correspond to dense subnetworks (Srihari and Leong, 2013). Moreover, it has been found that some protein complexes are sparse, either due to the incompleteness of PPI networks or the nature of the complex composition (Srihari and Leong, 2012). For instance, examination of the protein complexes in yeast has pointed out that they vary in terms of density, ranging from star-shaped (Fig. 1A) to the more dense complexes (Fig. 1B) including those that form cliques (Fig. 1C). This observation has led to the design of algorithms to identify sparse (Srihari and Leong, 2012; Yong *et al.*, 2012) as well as small complexes (Ruan *et al.*, 2018; Yong *et al.*, 2014), which have slightly improved the recall of protein complexes.

This brief summary of the state-of-the-art approaches for protein complex prediction based on PPI networks points at the need to formalize the problem of identifying protein complexes so that both sparse and dense subnetworks can be considered. Moreover, the existing network clustering approaches depend on multiple parameters, and it is challenging to optimize their values on different network topologies and gold standards. Further, the optimal values of different parameters may result in drastically different predictions of protein complexes. Therefore, it is critical that the formalization of the problem of protein complex prediction is parameter-free. It is also important to ensure that the novel formalization extracts clusterings of local structures with large cluster quality measures. This will allow us to determine the effect of the network structure on the prediction of protein complexes.

Our contribution is fourfold: (i) we formalize the concept of a protein complex as a biclique spanned subgraph, that addresses the density issue of the existing approaches; (ii) we propose a parameter-free approximation algorithm, termed Protein Complexes from Coherent Partition (PC2P), that solves the network partitioning into biclique spanned subgraphs by removing the minimum number of edges in a given PPI network; (iii) we demonstrate that the resulting clusterings are of high modularity, thus reflecting the local structures in the network and (iv) we provide a thorough comparison with nine seminal unsupervised approaches for protein complex prediction and show that our biclique spanned subgraph partition outperforms them with respect to twelve established performance measures and composite score combining five of these measures.

## 2 Materials and methods

### 2.1 Contending algorithms

We compared the performance of our approximation algorithm with nine contenders, including: Markov Clustering (MCL) (Enright *et al.*, 2002), Molecular Complex Detection (MCODE) (Bader and Hogue, 2003), CFinder (Adamcsek *et al.*, 2006), Affinity Purification (AP) (Frey and Dueck, 2007), CMC (Liu *et al.*, 2009), Clustering with overlapping neighborhood extension (ClusterOne) (Nepusz *et al.*, 2012), Core and Peel (Pellegrini *et al.*, 2016), Inter Module Hub Removal Clustering (IMHRC) (Maddi and Eslahchi, 2017) and ProRank+ (Hanna and Zaki, 2014). We prioritized the inclusion of approaches which have implementations in the public domain and do not rely on availability of additional knowledge (e.g. ontologies), to facilitate fair comparison. For AP, MCL and ClusterOne, we used the implementation available in Cytoscape, while for CMC, CFinder and ProRank+, we download the respective software from https://www.comp.nus.edu.sg/∼wongls/projects/complexprediction/CMC-26may09/, http://cfinder.org/ and https://faculty.uaeu.ac.ae/nzaki/Research.htm. Similarly, we used the implementation of Core and Peel and IMHRC which are available at http://bioalgo.iit.cnr.it/ppin/index.php?page=app&id=572664770 and http://www.eslahchilab.ir/softwares/cdap, respectively. Since all of these approaches depend on multiple parameters, we have used their default values. Optimizing the parameters is challenging since they depend on both the networks and gold standards used as well as on the objective to be optimized. Finally, optimization of different performance measures yields different clusterings (i.e. predicted protein complexes), rendering it impossible to do meaningful interpretation and combination of the findings.

### 2.2 Networks and gold standards used

To test our proposed approach and compare its performance with contending algorithms, we used PPI networks and gold standards of protein complexes from yeast and human. For yeast, we used four PPI networks, including: Collins (Collins *et al.*, 2007), Krogan core, containing highly reliable interactions (probability ≥ 0.273), Krogan extended, containing more interactions of smaller reliability (probability ≥ 0.101) (Krogan *et al.*, 2006) and Gavin (Gavin *et al.*, 2006). The gold standard was given by the CYC2008 (Pu *et al.*, 2009), an update to The Munich Information Centre for Protein Sequences (MIPS) catalogue (Mewes, 2004), and complexes derived from the Saccharomyces Genome Database (SGD) (Hong *et al.*, 2007). In the case of human, we used the up-to-date PPI networks obtained from STRING (Szklarczyk *et al.*, 2015) and PIPS (McDowall *et al.*, 2009) databases. The cut-off score for the STRING and PIPS PPI networks are set to 999 and 25, respectively. In addition, we employed CORUM as gold standard for human protein complexes (Giurgiu *et al.*, 2019). The used PPI networks and gold standards differ with respect to the number of proteins and interactions they include. For completeness, Supplementary Table S1 includes these features for the PPI networks, gold standards, and their intersections employed in the analyses.

### 2.3 Performance measures

The predicted complexes based on the clusterings obtained from the computational approaches, above, were compared with the complexes in the gold standard based on twelve performance measures: sensitivity, positive predictive value, accuracy and separation from Brohée and van Helden (2006), fraction match and maximum matching ratio from Nepusz *et al.* (2012), precision, recall and F-measure from Liu *et al.* (2009) and precision^+^, recall^+^ and F-measure^+^ from Maddi *et al.* (2019). To define the first three measures, a contingency table T was assembled with n rows denoting complexes and m columns representing clusters. The entry $t_{i,j}$ contains the number of shared proteins between complex i and cluster j. Let $N_{i}$ denote the number of proteins in complex i, $N=\sum_{i}N_{i}$ and $P_{j}$ stand for the number of proteins in cluster j. Since some of the compared algorithms allow for overlapping clusters, the marginal row and column sums may not correspond to the size of complexes and clusters, respectively.

The sensitivity (SN) of a clustering is defined as *SN* $=\frac{\sum_{i}\underset{j}{\max}t_{i,j}}{\sum_{i}N_{i}}$, while the positive predictive value (PPV) is given by $\mathrm{PPV}=\frac{\sum_{j}\underset{i}{\max}t_{i,j}}{\sum_{j}\sum_{i}t_{i,j}}$. The accuracy (ACC) is then given by the geometric mean of sensitivity and positive predictive value, i.e. $\mathrm{ACC}=\sqrt{SN\cdot \mathrm{PPV}}$. Separation (SEP) of a clustering is defined as $=\sqrt{\frac{1}{nm}\sum_{i}\sum_{j}\left(\frac{t_{i,j}}{t_{.j}}.\frac{t_{i,j}}{t_{i.}}\right).\sum_{j}\sum_{i}\left(\frac{t_{i,j}}{t_{.j}}.\frac{t_{i,j}}{t_{i.}}\right)}$, whereby the product of the proportion of proteins of a complex that are found in a cluster and the proportion of proteins in a cluster that are found in a complex, to quantify a relation between clusters and complexes. The fraction match (FRM) for a complex i and cluster j is given by $\frac{t_{i,j}^{2}}{N_{i}P_{j}}$ and we considered the fraction of clusters of FRM greater than 0.25, as suggested by Nepusz *et al.* (2012). Finally, the maximum matching ratio (MMR) is given by the value of the maximum matching per complex in a bipartite graph, with nodes corresponding to the gold standard complexes and extracted cluster, as two partitions, and edges denoting the overlap between the respective complexes and clusters.

To calculate precision and recall, we first determine if any of the predicted clusters matches with any of the reference complexes. Following Habibi *et al.* (2010), Liu *et al.* (2009) and Srihari *et al.* (2015), we used Jaccard similarity (i.e. $\mathrm{Jaccard}(P,C)\;=\;\frac{\left|P\cap C\right|}{\left|P\cup C\right|}$). The predicted cluster is said to match the reference complex if their Jaccard similarity is greater than 0.5. The precision is defined as a ratio between the number of predicted clusters that match the reference complexes and the number of predicted clusters ($\mathrm{Precision}\;=\;\frac{\left|\left\{p_{i}\epsilon P|\;\exists c_{j}\epsilon C,\;p_{i}\;\mathrm{matches}\;c_{j}\right\}\right|}{\left|P\right|}$). The recall is defined as the ratio between the number of reference complexes that match the predicted complexes and the total number of reference complexes ($\mathrm{Recall}\;=\;\frac{\left|\left\{c_{i}\epsilon C|\;\exists p_{j}\epsilon P,\;p_{i}\;\mathrm{matches}\;c_{j}\right\}\right|}{\left|C\right|}$). Finally the F-measure is defined as$\;\frac{2\cdot \mathrm{Precision}\cdot \mathrm{Recall}}{\mathrm{Precision}+\mathrm{Recall}}$. The measure precision^+^ is given by $\frac{N_{P}^{+}}{\left|P\right|}$ and$\;N_{P}^{+}=\left|\left\{p_{i}\epsilon P|\;\exists c_{j}\epsilon C,\;\mathrm{NA}(p_{i},\;c_{j})\geq \theta ,(p_{i},\;c_{j})\in \mathrm{Match}(P,C,\theta )\;\right\}\right|$. Similarly, recall^+^ is defined as $\frac{N_{C}^{+}}{\left|C\right|}$ and$\;N_{C}^{+}=\left|\left\{c_{i}\epsilon C|\;\exists p_{j}\epsilon P,\;\mathrm{NA}(p_{i},\;c_{j})\geq \theta ,(p_{i},\;c_{j})\in \mathrm{Match}(P,C,\theta )\;\right\}\right|$. In the definitions of these terms$,\;\mathrm{NA}(p_{i},\;c_{j})\;$represents the neighborhood affinity score between complexes and clusters, while Match(P,C,θ) includes the edges by employing maximum matching algorithm on the bipartite graph that has complexes on one side and the clusters on the other side. We note that θ is a parameter that can take values in the interval [0, 1]. These measures were selected based on their usage in seminal studies about prediction of protein complexes (Adamcsek *et al.*, 2006; Liu *et al.*, 2009; Nepusz *et al.*, 2012; Pellegrini *et al.*, 2016). Further, the composite score is given by the sum of the values for MMR, FRM, SEP, ACC and F-measure (Cao *et al.*, 2018; Nepusz *et al.*, 2012; Wang *et al.*, 2018). Finally, as suggested in Maddi *et al.* (2019), the sum of MMR and F-measure^+^ over different threshold values was utilized as another performance measure.

Functional similarity between two proteins can be assessed by semantic similarity of their respective GO annotation terms (Cho *et al.*, 2007). We employed the GOSim R package (Fröhlich *et al.*, 2007) to determine similarity between protein pairs in a given cluster. The semantic similarity of a cluster was summarized by the mean and minimum of the semantic similarity of the protein pairs in the cluster, respectively, and their performance was compared. Moreover, the biological relevance of predicted complexes is evaluated by enrichment analysis of the biological process, molecular function and cellular component terms. We applied the clusterProfiler R package (Yu* et al.*, 2012) to obtain the biological relevance of predicted complexes. The enrichment score of each GO category was computed for all clusters and their performance was compared.

Further, structural quality of a network clustering was assessed by the widely used measure of modularity (Brandes *et al.*, 2008). The modularity of a clustering C is given by $q\left(C\right)=\sum_{C\in C}\left[\frac{\left|E\left(C\right)\right|}{m}-{\left(\frac{\sum_{v\in C}\deg\left(v\right)}{2m}\right)}^{2}\right]$, where E(C) denotes the number of edges with both ends in the cluster C in C, deg⁡(v) denotes the degree of v, and m is the number of edges in the graph. However, modularity may not be suitable to compare algorithms which result in overlapping clusters (Lázár *et al.*, 2010). Therefore, we also measured structural cluster quality as defined by (Lázár *et al.*, 2010), which accounts for overlapping clusters.

## 3 Results

### 3.1 Protein complexes as biclique spanned subgraphs

We formalize the concept of a protein complex by a biclique spanned graph. A graph G=(V,E) is biclique spanned if the node set can be partitioned into two subsets, $V_{1}(G)$ and $V_{2}(G)$, with $V_{1}\left(G\right)\cap V_{2}\left(G\right)=\emptyset$, $V_{1}\left(G\right)\cup V_{2}\left(G\right)=V(G)$, such that E(G) contains the edges (u,v) for every $u\in V_{1}(G)$ and every $v\in V_{2}(G)$ as well as additional edges between the nodes of each partition. As a result, biclique spanned graphs allow for modelling both sparse graphs (e.g. stars) and dense graphs (e.g. bicliques and cliques). The distance between any two nodes in a biclique spanned graph is at most 2. It can be shown that a graph G is biclique spanned if and only if its complement, $\overset{-}{G}$, is disconnected (i.e. it contains more than one connected component) (Akiyama and Harary, 1981). Thus, the concept of a biclique spanned graph provides a natural formalization of network cluster based on connectedness, since the complement of a cluster in a real-world network is expected to be disconnected. For instance, all graphs in Figure 1 are biclique spanned, including stars as well as cliques.

A coherent network partition in a graph G is a partition $C=\{C_{1},\ldots ,\;C_{k}\}$ of the node set V(G) such that every $C_{i}$, 1≤i≤k, induces disconnected subgraph in $\overset{-}{G}$, i.e. a biclique spanned subgraph in G (Angeleska and Nikoloski, 2019). A network partition C is obtained by removing all edges (u,v), $u\in C_{i}$, $v\in C_{j}$, 1≤i≠j≤k, resulting in the clusters $C_{1},\ldots ,\;C_{k}$. The edges that render a network partition C form an edge cut E(C). A coherent network partition C is optimal if it minimizes the number of edges in the edge cut E(C) that renders the partition.

Given a graph G that represents a PPI network, we hypothesize that an optimal coherent network partition corresponds to partitioning of the network into protein complexes. It has recently been shown that the problem of finding an optimal coherent network partition is NP-hard (Angeleska and Nikoloski, 2019). Thus, in the following, we present a greedy approximation algorithm for solving this optimization problem, in which iteratively identify the node of the best score, defined below, together with the biclique spanned subgraph in which it participates followed by its removal from the network.

### 3.2 PC2P—an algorithm to predict protein complexes

Given a graph G, our approach, termed Protein Complexes from Coherent Partitions (PC2P), for every node u determines a score that quantifies the quality of a biclique spanned subgraph in the second neighborhood of u, denoted by $N_{2}(u)$. We consider $N_{2}(u)$, that contains all nodes at distance at most 2 from u, since a biclique spanned subgraph that includes u is necessarily of diameter 2. The score is given by: $\frac{\left|E_{\mathrm{out}}\right|}{\left|E_{in}\right|}\frac{1}{d}$, in which d is the density of the biclique spanned subgraph in $N_{2}(u)$, and is computed as $\frac{2\left|E_{in}\right|}{\left|V\right|\left(\left|V-1\right|\right)}$, where $\left|E_{\mathrm{out}}\right|$ is the number of edges connecting the subgraph to the rest of the network, $\left|E_{in}\right|$ is the number of edges inside the subgraph and $\left|V\right|$ is the number of nodes in the subgraph. It aims at finding a compromise between the number of edges removed to obtain the biclique spanned subgraph and edges that remain in it. PC2P then selects the node with the smallest score and removes the biclique spanned subgraph in $N_{2}(u)$ from the graph. The procedure is repeated as long as there are connected components in G (Algorithm 1).

To obtain a biclique spanned subgraph in $N_{2}(u)$ which minimizes the proposed score (Algorithm 2), we first investigate the complement of $N_{2}(u)$. If the complement of $N_{2}(u)$ is disconnected, then $N_{2}(u)$ forms a biclique spanned subgraph; otherwise, we seek to identify the minimum node cut in the complement of $N_{2}(u)$, i.e. the smallest number of nodes whose removal render the subgraph disconnected. The minimum node cut can be determined by solving a series of max-flow min-cut problems (Kanevsky, 1993). To speed up the calculations, we check if the complement of the second neighborhood $N_{2}(u)$ contains an articulation point, in which case the removal of a single node disconnects the graph. Moreover, since the minimum node connectivity of a graph is at least as large as the minimum degree (Kammer and Täubig, 2004), to render the algorithm more efficient, we remove the neighbors of a node of minimum degree which yields the smallest score.

**Algorithm 1 PC2P algorithm**

1: **procedure** Find_Coherent_Partition (G)

2: *edge_cut* ←∅

3: connected_*components* ← connected component of G

4: **while** there is a component in *connected_components* **do**

5:    **for** each node v in *connected_component* **do**

6:       *result* ← CNP(v*,connected_components*)

7:    Update *edge_cut*

8:    Update *connected_components*

9: **return** (*edge_cut*)

**Algorithm 2 CNP Function**

1: **procedure** CNP (v, *cmp*)

2: $N_{2}\leftarrow$ second neighborhood of node v in *cmp*

3: *complement_*$N_{2}\leftarrow$ complement of $N_{2}$

4: **if** *complement_*$N_{2}$ is **disconnected then**

5:   *cut_ratio* ← calculate cut ratio for the *cmp*

6:   **return** (*cmp, cut_ratio*)

7: **else**

8:   *cut_node* ← find minimum node cut in *complement_*$N_{2}$

9:   **remove** *cut_node* from *cmp*

10:  *cut_ratio* ← compute cut ratio for the *cmp* sub-graph

11:  **return** (*cmp, cut_ratio*)

The complexity of finding a complement of a graph on n nodes is in $O(n^{2})$, finding an articulation point can be achieved in time O(n+m), identifying the minimum degree node is in O(n), while determining the neighbors of the nodes of minimum degree that result in the smallest score is in $O(n^{2})$. Since the second neighbor $N_{2}(u)$ contains at most $O(\Delta^{2})$ nodes, where Δ is the maximum node degree in the graph, the complexity of one iteration of the algorithm is in $O(n\Delta^{4})$. As a result, if in each round the algorithm finds a component with $\Delta^{2}$ nodes, then the algorithm will finish after $\frac{n}{\Delta^{2}}$ iterations. Therefore, the time complexity of the sequential algorithm is in $O(n^{2}\Delta^{2})$ and the parallel version of the algorithm, which renders it faster by a factor of P, which is the number of processors $O(\frac{n^{2}}{P}\Delta^{2})$.

There are two other algorithms for graph clustering based on iterative determination of min cuts, but they do not operate in the complement of a given graph and have not yet been applied to the problem of protein complex prediction. The algorithm of Flake *et al.* (2004) aims at identifying clusters in given graph by determining its min-cut tree (Gomory and Hu, 1961) and ensuring that the inter-cluster and intra-cluster edge capacities are bounded from above and below by a user-specified parameter α. To this end min-cuts are iteratively determined in the graph augmented by a sink node; the sink is connected to all other nodes by edges of capacity α. In contrast to PC2P, this algorithm is parameter-dependent and cannot identify biclique spanned subgraphs. Further, Hartuv and Shamir‘s algorithm depends on iterative determination of min-cuts to arrive at highly connected subgraphs, whose connectivity is greater than n/2, with n denoting the number of nodes (Hartuv and Shamir, 2000). In contrast to PC2P, which can identify both sparse and dense clusters, the approach of Hartuv and Shamir identifies only dense clusters, in which the number of edges that grows quadratically with the number of nodes. In addition, this algorithm requires the knowledge of the entire graph, whereas ours is a local algorithm as it operates on the second neighborhood of nodes. Furthermore, while the generated highly connected clusters, like biclique spanned graphs, are of diameter no greater than two, the algorithm has the tendency to break up a sparse cluster into singletons. The latter issue is addressed by different heuristic steps (pre- and post-processing) applied on the obtained clusters (e.g. merging of singletons, removal of nodes of small degree), which also require user input.

We inspected the empirical running time on the four PPI networks of yeast and the two PPI networks of human, as specified in the methods (see Supplementary Table S5). We found that the running time scales with the diameter of the network. For instance, the KroganCore graph has a diameter of 12, while that of Collins is 15 in a smaller graph regarding the number of nodes; as seen in Supplementary Table S5, the running time for the former is 4 h, while for the latter it is 8 min. All mentioned experiments were carried on the same machine, an Intel(R) Xeon(R) CPU E5-2670 v2 with 2.50 GHz.

### 3.3 Structural, functional quality and biological relevance of PC2P clusters

**Fig. 2.**
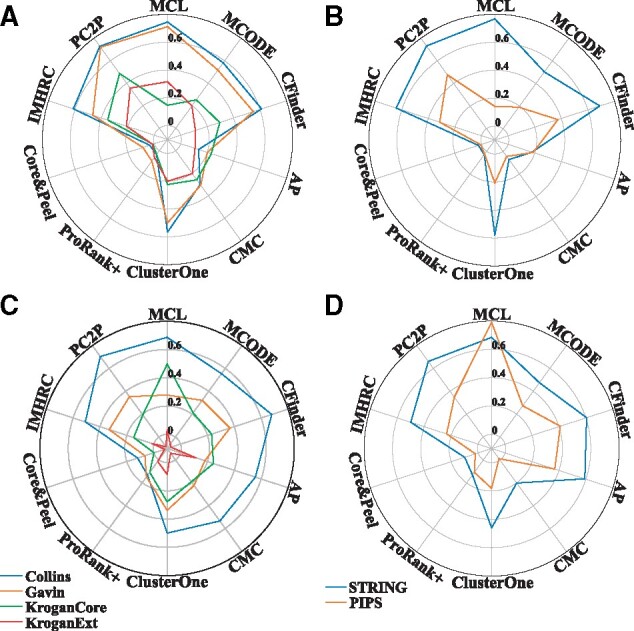
Comparison of modularity and cluster quality measure of predicted protein complexes. Nine contending algorithms were compared with PC2P with respect to the modularity and the cluster quality measure (Lázár *et al.*, 2010) of the clusterings, resulting in protein complexes, on (**A**) and (**C**) four PPI networks in yeast and (**B**) and (**D**) two PPI networks in human. PCP2 has the highest modularity compared to all contending algorithms in three out of four and one out of two networks of yeast and human respectively. Whereas it outperforms other contenders only in one of the yeast networks when cluster quality measure is considered

To inspect the performance of PC2P with respect to structural, functional quality and biological relevance of the resulting clusters, we determined the modularity, the GO semantic similarity and standard enrichment score of the clusterings in four PPI networks for yeast, i.e. Collins, Gavin, KroganCore and KroganExt, and two for human, i.e. STRING and PIPS. We compared the performance of nine other approaches, i.e. MCL, MCODE, CFinder, AP, CMC, ClusterOne, ProRank+, Core&Peel and IMHRC (see Section 2.1) with respect to these three performance measures (see Section 2.3).

or the yeast PPI networks, we found that PC2P results in the largest modularity with respect to nine contenders for three out of four networks irrespective of whether or not cluster overlap was considered (Fig. 2A, Supplementary Table S2). For the Collins PPI network our approach ranked second, with ∼2% decrease in modularity compared to MCL. Similarly, for the PPI networks of human, PC2P outperformed all approaches on the PIPS network. Moreover, it ranked second for the STRING network, with ∼ 4% decrease in modularity in comparison to MCL (Fig. 2B, Supplementary Table S2). For fair comparison with approaches that result in overlapping clusters, we also used the cluster quality measure defined in Lázár *et al.* (2010). We found that PC2P outperformed the other contending approaches on the Collins PPI, and it ranked second with ∼1% smaller value than CFinder on the Gavin PPI network of yeast (Fig. 2C and D, Supplementary Table S2). For KroganCore and KroganExt, MCL and AP were the best ranked approaches with respect to this measure. We found that MCL showed the highest modularity of overlapping clusters in human and PC2P ranked second for STRING network, with ∼2% decrease in modularity (Fig. 2C and D, Supplementary Table S2).

We also calculated the modularity of the clusterings obtained when only the proteins in two yeast gold standard datasets of protein complexes, CYC2008 and SGD, were considered (Supplementary Table S1). Our findings demonstrated that the modularity of PC2P clusterings was the largest in 1 out of 8 combinations of PPI networks and gold standards. In all but one of the remaining cases, PC2P resulted in the second best performance, closely behind MCL (Supplementary Table S3). For the human gold standard of protein complexes, CORUM, PC2P clusterings were of the highest modularity for the two PPI networks (Supplementary Table S3). Interestingly, PC2P outperformed one of the compared approaches when the cluster quality measure from Lázár *et al.* (2010) was used on the combination of PPI networks and gold standards of yeast. In the remaining combinations, PC2P ranked among the top three performers, except in the case of KroganExt PPI where all approaches resulted in small values for the modularity of overlapping clusters. MCL showed the best performance with respect to this measure across all combinations of human PPI networks and gold standard, however, PC2P ranked second (Supplementary Table S3). Altogether, these findings indicated that PC2P can effectively identify the modular structure in protein interaction network as a prerequisite for prediction of protein complexes.

**Fig. 3.**
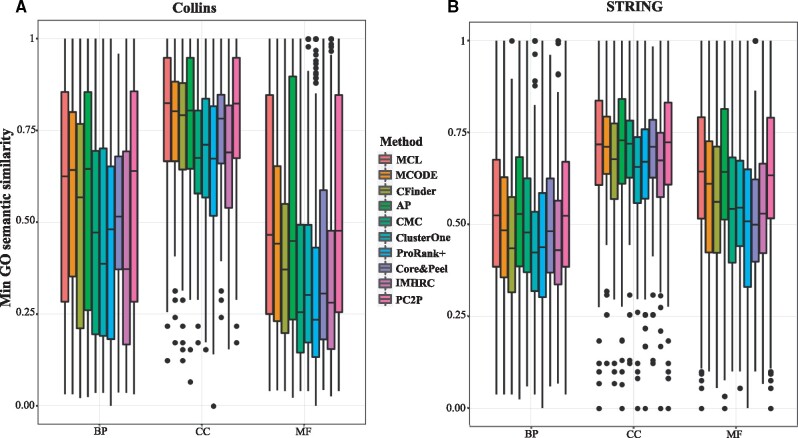
Comparison of minimum GO semantic similarity for predicted protein complexes. PCP2 is compared against nine algorithms (ordered by the year of publication) with respect to the distribution of minimum GO semantic similarity over all clusters, for (**A**) Collins PPI network of yeast and (**B**) STRING PPI network of human. GO semantic similarity is determined for the three GO categories: biological process (BP), cellular component (CC) and molecular function (MF)

With respect to the functional quality of the clusterings, we determined the minimum and mean semantic similarity for every pair of proteins in each predicted complex (i.e. clusters) and compared the distribution of these values over all clusters for the nine contenders. For the minimum GO semantic similarity, in the four yeast PPI networks as well as in the two human PPI networks, we found that all of the approaches resulted in largely overlapping distributions of GO semantic similarity over the respective clusters for the three GO categories, i.e. biological process (BP), cellular component (CC) and molecular function (MF) (Fig. 3A). Nevertheless, PC2P exhibited the largest median GO semantic similarity with respect to MF in two out of four yeast PPI networks and ranked second in the rest of PPI networks. While proteins involved in complexes may have distinct functions, they usually are annotated with multiple MF GO terms with indicate their involvement in the function of the complex as a whole, leading to larger GO semantic similarity (Ashburner, 2000). In the rest of the cases, PC2P showed comparable distributions and medians of GO semantic similarity to those of MCL. Similar findings were obtained for the two PPI networks of human (Fig. 3B).

Interestingly, the same conclusions can be drawn when analysing the distributions of mean GO semantic similarity over all clusters: all approaches, except for AP, exhibited comparable distributions, with the medians of Core&Peel and PC2P ranked highest (Supplementary Fig. S1). These findings hold for both yeast and human PPI networks, demonstrating that PC2P performs as well as the existing approaches in capturing functional quality of the resulting clusters.

We also conducted ANOVA analyses to test if PC2P results in smaller average GO semantic similarity in comparison to the contending algorithms. To summarize the GO semantic similarity of a cluster we considered the minimum and the mean semantic similarities. For the ANOVA with the minimum semantic similarity of each cluster, from 6 comparisons in each of the 4 yeast and 2 human PPI networks for the MF GO category PC2P has either the same or greater average in comparison to the other approaches. For the BP and CC GO categories, PC2P exhibits a smaller average in 1.85% and 0.37%of the comparisons, respectively, i.e. BP semantic similarity of Core&Peel and ProRank+ on Gavin and KroganExt and MCODE on the KroganCore PPI network as well as CC semantic similarity of Core&peel on KroganCore PPI networks (Supplementary Table S4). For the rest of comparisons, PC2P showed either higher or the same average as the other contenders. For the ANOVA with the mean semantic similarity for each cluster, PC2P exhibited either the same or greater average for the MF GO category. For the BP and CC GO categories, PC2P has a smaller average in 2.6% and 1.1% of the comparisons, correspondingly, i.e. BP semantic similarity of MCODE, ProRank+ and Core&Peel on KroganCore and ProRank+ and Core&Peel on Gavin and KroganExt as well as CC semantic similarity of Core&Peel on Gavin, KroganCore and KroganExt (Supplementary Table S4). In the remaining comparisons, PC2P demonstrated better or the same average as the other methods. Altogether, these findings imply that PCP2 maintains the semantic similarity of the predicted protein complexes, in line with biological expectations.

Concerning biological relevance, we determined the enrichment score of the annotations for each predicted complex (i.e. cluster). We used the enrichGo function in the clusterProfiler package and selected Benjamini-Hochberg as a *P*-value adjustment method with a significant level of 0.05. We compared the average of predicted complexes with at least one enriched annotation over all clusters for the nine approaches across all datasets (Supplementary Table S6). The GO annotations with Inferred from electronic annotations (IEA), No biological Data available (ND), Non-traceable Author Statement (NAS) and Inferred from Physical Interaction (IPI) evidence codes were dropped from the computation. The results illustrate that PC2P predicts biologically relevant clusters with enrichment scores that are of moderate value that ranks in the top 50% of the contenders with respect to the different GO categories (Supplementary Table S6).

### 3.4 PC2P outperforms contending approaches for protein complex prediction

Next, we employed the two gold standards for yeast and the one for human with the respective PPI networks to compare the performance of PC2P with respect to its ability to accurately predict protein complexes. To this end, we determined twelve performance measures, including: maximum matching ratio (MMR), fraction match (FRM), separation (SEP), positive predictive value (PPV), Sensitivity (SN), accuracy (ACC), precision, recall, F-measure, precision^+^, recall^+^ and F-measure^+^ (see Section 2.2). In addition, we calculated a composite score that combines MMR, FMR, SEP, ACC and F-measure for each of the ten compared approaches. Furthermore, we calculated the MMR and F-measure^+^ over different θ values and used their sum as an additional score.

**Fig. 4.**
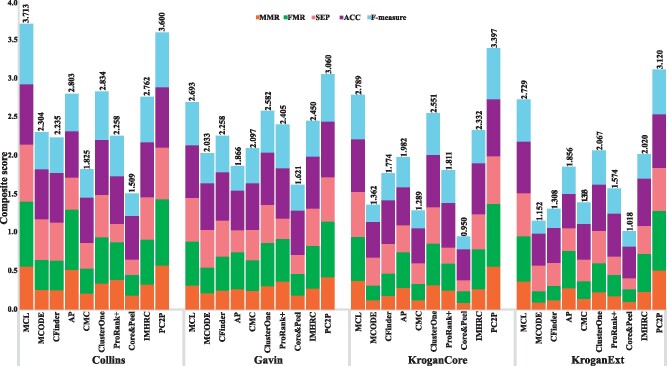
Comparative analysis of approaches for prediction of protein complexes in yeast. The comparative analyses is conducted with respect to a composite score combining five performance measures, maximum matching ratio (MMR), fraction match (FMR), separation (SEP), accuracy (ACC) and F-measure. Ten approaches, ordered by the year of publication, are compared on four PPI networks in yeast with respect to CYC2008 gold standard. PCP2 outperforms all approaches on three of the four networks

For the 8 combinations of protein interaction networks and gold standards of protein complexes in yeast, our findings demonstrated that PC2P exhibited the highest MMR and FRM values in all cases as well as the highest ACC, PPV and recall^+^ in 7 combinations. Moreover, PC2P resulted in the highest recall in all 8 combinations, as high as 71% and 69% in Collins and KroganCore PPI networks with CYC2008 gold standard (Supplementary Fig. S2, Supplementary Table S3). It is then not surprising that PC2P outperformed all contenders with respect to the composite score in 7 out of 8 combinations for yeast (Fig. 4, Supplementary Table S3).

**Fig. 5.**
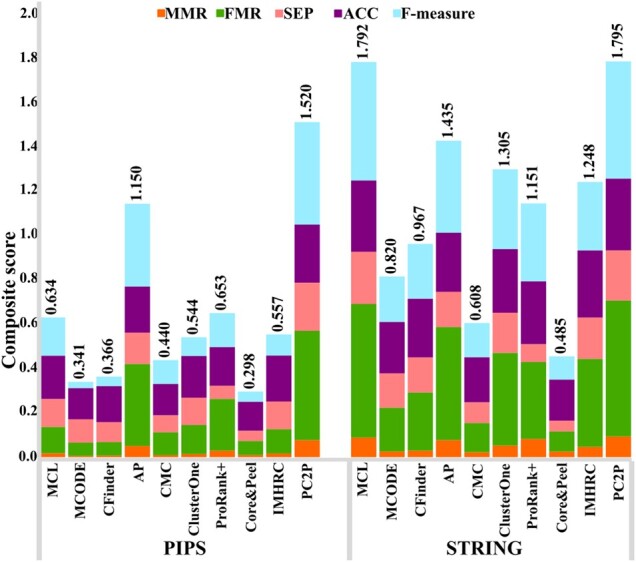
Comparative analysis of approaches for prediction of protein complexes in human. The comparative analyses is conducted with respect to a composite score (see caption of Fig. 4). Ten approaches, ordered by the year of publication, are compared on two PPI networks with respect to CORUM gold standard. PCP2 outperforms all approaches

**Fig. 6.**
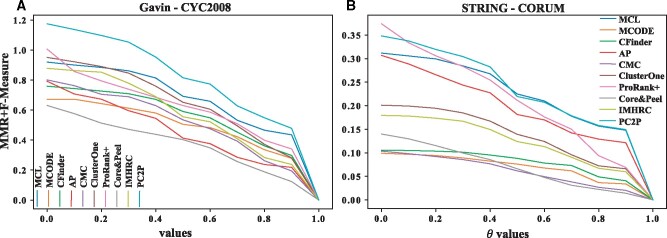
Summation of MMR and F-measure+ of approaches for prediction of protein complexes. The sum of MMR and F-measure+ is determined over the range of values for θ from 0 to 1. Ten approaches, ordered by the year of publication, are compared on two PPI networks with respect to CYC2008 and CORUM gold standard for (**A**) Gavin and (**B**) STRING in yeast and human respectively. PCP2 outperforms all compared approaches

Similarly, MMR, FMR, ACC and recall were the largest for PC2P in the 2 combinations of protein interaction networks with the gold standard of protein complexes in human (Fig. 5, Supplementary Table S3). The second and third best performers in the case of both yeast and human included MCL and ClusterOne. Further, we considered the sum of the MMR and F-measure^+^ values over the range of θ values from 0 to 1 for all combination of PPI networks and gold standards in human and yeast as another way of ranking (Maddi *et al.*, 2019). Here, too, PC2P exhibited the highest values for different values of θ for all combinations except for Collins PPI network in yeast. Similarly, PC2P exhibited the highest values over the considered range of θ for the human PIPS as well as STRING PPI network (Fig. 6, Supplementary Fig. S3). Therefore, our findings demonstrated that PC2P outperforms the contending approaches for protein complex prediction in both yeast and human PPI networks.

## 4 Discussion

Assembly of high-quality gold standards of protein complexes in model eukaryotes has pointed out that protein complexes have diverse structure that may not be accurately represented by dense graphs. Nevertheless, the existing unsupervised approaches for prediction of protein complexes from PPI networks rely on partitioning the network into dense subgraphs, which affects their performance. These approaches also suffer from very low recall, and attempts have been made to resolve these issues by designing of algorithms that specifically seek to identify sparse structures.

Here, we offered a new perspective on the prediction of protein complexes from PPI networks by formalizing a protein complex as a biclique spanned graph. This allowed us to model protein complexes on the continuum from sparse to dense graphs, since the class of biclique spanned graphs includes stars, bicliques as well as cliques as special subclasses. This is the major contribution of our formalization, which we later show that it also leads to improvement of recall over the existing solutions. Further, such a formalization facilitated casting of the problem of protein complex prediction as that of finding an optimal coherent partition. Since this problem is NP-hard (Angeleska and Nikoloski, 2019), we proposed a greedy approximation algorithm called PC2P, for protein complexes from coherent partition. In contrast to the existing algorithms, PC2P is parameter-free and, therefore, can be employed objectively, without user-specified and case-to-case tuned parameters. This is another major improvement over the existing solutions for determining protein complexes in large-scale PPI networks.

Extensive comparative analyses demonstrated that PC2P outperformed state-of-the-art contending approaches with respect to seminal performance measures in PPI networks from yeast and human, while ensuring that the overall semantic similarity of the predicted proteins is high, in line with biological expectation. Most importantly, PC2P resulted in the largest recall (and refinements of this measure) in majority of examined datasets, demonstrating that PC2P offers a parameter-free means to overcome the key shortcoming of the existing approaches. Finally, we also observed that the distribution of cluster sizes resulting from PC2P does not differ with respect to the compared approaches: For all algorithms, except ClusterOne, Core&Peel and IMHRC, the cluster size distributions are monotonically decreasing (Supplementary Fig. S4). This finding indicates that the improvements of performance in predicting protein complexes by PC2P are due to the formalization of the protein complex as a biclique spanned subgraph rather than the resulting distribution of cluster sizes.

Future studies will aim on one hand to speed up the running time of the implementation of the algorithm, by exploring the properties of the second neighborhood of a node; on the other, additional research efforts will be aimed to identify the connection between single gene and whole genome duplication in driving the formation of biclique spanned subgraphs underlying the predicted protein complexes. Finally, we note that the current formulation of PC2P is not applicable for detection of overlapping clusters and weighted networks, and future work will address these shortcomings to improve the versatility of the presented formulation of protein complexes in terms of bicliques.

## Supplementary Material

btaa1089_Supplementary_Data
