# Supplementary material for: PC2P: parameter-free network-based prediction of protein complexes
Source: Bioinformatics. 2021 Jan 8;37(1):73–81. doi: 10.1093/bioinformatics/btaa1089 (PMC8034538; doi:10.1093/bioinformatics/btaa1089)

Supplementary Table 1. Shown are the properties, i.e. number of proteins and interactions, in the PPI networks used in our analysis. Y stands for yeast and H for human. We also show the number of proteins and complexes in each of the gold standards used in our analysis. Finally, we also show the number of nodes and edges in the intersection of the PPI networks and gold standards of yeast and human, respectively.

PPI networks

	Collins (Y)	Gavin (Y)	KroganCore (Y)	KroganExt (Y)	STRING (H)	PIPS (H)
#Proteins	1,622	1,855	2,708	3,672	2,227	1,673
#Interactions	9,074	7,669	7,123	14,317	8,565	5,385
Diameter	15	13	12	10	21	18

Gold standards

	SGD(Y)	CYC2008(Y)	Corum
#proteins	1,279	1,627	4,479
#complexes	323	408	4,274
#complexes ≥ 3	238	236	2,783

Intersection of PPI networks and gold standards

MIPS	Collins(Y)	Gavin(Y)	KroganCore(Y)	KroganExt(Y)
#Proteins	773	766	780	888
#interactions	4,778	2,695	1,797	2,639

SGD	Collins(Y)	Gavin(Y)	KroganCore(Y)	KroganExt(Y)
#Proteins	830	776	944	1,057
#interactions	2,955	2,639	2,554	3,688

CYC2008	Collins(Y)	Gavin(Y)	KroganCore(Y)	KroganExt(Y)
#Proteins	1,116	1,047	1,149	1,263
#interactions	6,392	4,031	3,169	4,714

Corum	STRING(H)	PIPS(H)
#Proteins	1,711	871
#interactions	7,289	2,692

Supplementary Table 2. Shown is the modularity of the clustering obtained by applying each of the eight compared algorithms on the PPI networks from yeast and human, respectively. The algorithms are ordered based on the year of publication.

Modularity-Yeast

Method	Collins	Rank	Deviation of second best compared to PC2P	Gavin	Rank	Deviation of second best compared to PC2P	KroganCore	Rank	Deviation of second best compared to PC2P	KroganExt	Rank	Deviation of second best compared to PC2P
MCL	0.7450	1	Not Best 2.2%	0.7115	2	1.12%	0.1461	8	27.63%	0.3157	2	11.48%
MCODE	0.5817	5		0.5166	4		0.2522	5		0.1982	6	
CFinder	0.6116	4		0.5487	3		0.2953	3		0.1108	7	
AP	0.1350	8		0.2017	8		0.2314	6		0.1079	8	
CMC	0.3000	7		0.3065	7		0.2586	4		0.2040	4	
ClusterOne	0.5671	6		0.5003	5		0.2244	7		0.2005	5	
ProRank+	0.0341	10		0.0917	9		0.0338	9		0.0603	9	
Core&Peel	0.0346	9		0.0862	10		0.0102	10		0.0132	10	
IMHRC	0.6125	3		0.4652	6		0.3527	2		0.2107	3	
PC2P	0.7287	2		0.7196	1		0.4873	1		0.3566	1	

Modularity-Human

Method	STRING	Rank	Deviation of PC2P Compared to the Best	PIPS	Rank	Deviation of second best compared to PC2P
MCL	0.7664	1	Not Best 4.56%	0.1413	7	21.63%
MCODE	0.5024	6		0.1908	5	
CFinder	0.6893	3		0.3742	2	
AP	0.1798	7		0.1880	6	
CMC	0.0726	8		0.0442	8	
ClusterOne	0.5799	5		0.2074	4	
ProRank+	0.0287	9		0.0243	9	
Core&Peel	0.0233	10		0.0069	10	
IMHRC	0.6423	4		0.3142	3	
PC2P	0.7314	2		0.4774	1	

Supplementary Table 2. Shown is the modularity of the clustering obtained by applying each of the eight compared algorithms on the PPI networks from yeast and human, respectively. The algorithms are ordered based on the year of publication.

Overlapped Modularity-Yeast												
Method	Collins	Rank	Deviation of second best compared to PC2P	Gavin	Rank	Deviation of second best compared to PC2P	KroganCore	Rank	Deviation of second best compared to PC2P	KroganExt	Rank	Deviation of second best compared to PC2P
MCL	0.6866	2	2.58%	0.2781	6	Not Best 3.77%	0.4969	1	Not Best 44.16%	0.0331	3	Not Best 26.69%
MCODE	0.5543	5		0.3211	5		0.2117	5		-0.0624	7	
CFinder	0.6783	3		0.3675	1		0.2307	4		-0.0713	8	
AP	0.5566	4		0.1804	8		0.2448	3		0.1145	1	
CMC	0.5347	6		0.2240	7		0.1911	7		-0.0588	6	
ClusterOne	0.4990	8		0.3381	3		0.2775	2		0.0839	2	
ProRank+	0.1347	9		0.1491	9		0.1090	9		0.0135	5	
Core&Peel	0.1193	10		0.0655	10		-0.0020	10		-0.0811	9	
IMHRC	0.5089	7		0.3313	4		0.1493	8		0.0149	4	
PC2P	0.7048	1		0.3536	2		0.1933	6		-0.0846	10	

Overlapped Modularity-Human						
Method	STRING	Rank	Deviation of PC2P Compared to the Best	PIPS	Rank	Deviation of second best compared to PC2P
MCL	0.6845	1	Not Best 3.58%	0.7908	1	Not Best 48.21%
MCODE	0.4742	6		0.2735	5	
CFinder	0.6050	3		0.4096	2	
AP	0.5916	4		0.3687	3	
CMC	0.2009	8		-0.0115	10	
ClusterOne	0.4603	7		0.1809	7	
ProRank+	0.1301	9		0.1078	8	
Core&Peel	0.0997	10		0.0208	9	
IMHRC	0.4999	5		0.2338	6	
PC2P	0.6600	2		0.3480	4	

Supplementary Table 3. Comparative analysis of eight algorithms for prediction of protein complexes with respect to nine performance measures as well as a composite score that combines five of them. Shown is also the modularity of the resulting clusterings.

Comparative analysis

Yeast - CYC2008

Data-set	Method	# Clusters	MMR	FRM	SEP	PPV	SN	ACC	Prec	Rec	F-measure	CDAP_prec	CDAP_rec	CDAP_fmeas	CDAP_F_MMR	Modularity	Overlapped Modularity	Composite Score (CS) MMR + FRM + SEP + ACC + F-measure	CS Ranking	Deviation of PC2P to Max in CS	Deviation of second best in CS compared to PC2P (if PC2P is best)
Collin	MCL	193	0.558	0.840	0.743	0.648	0.950	0.785	0.912	0.693	0.787	0.870	0.573	0.691	1.250	0.799	0.776	3.713	1	3.05%	Not Best
	MCODE	93	0.255	0.389	0.528	0.665	0.633	0.649	0.892	0.331	0.483	0.849	0.270	0.409	0.665	0.623	0.669	2.304	6		
	CFinder	89	0.247	0.389	0.493	0.532	0.775	0.642	0.888	0.314	0.464	0.843	0.256	0.393	0.640	0.749	0.770	2.235	8		
	AP	413	0.511	0.785	0.416	0.506	0.717	0.603	0.392	0.645	0.488	0.356	0.502	0.416	0.928	0.270	0.618	2.803	4		
	CMC	119	0.205	0.324	0.333	0.646	0.538	0.590	0.622	0.266	0.373	0.571	0.232	0.330	0.535	0.343	0.363	1.825	9		
	ClusterOne	127	0.338	0.597	0.553	0.611	0.833	0.714	0.898	0.488	0.632	0.819	0.355	0.495	0.833	0.705	0.636	2.834	3		
	ProRank+	458	0.382	0.491	0.238	0.477	0.796	0.616	0.683	0.433	0.530	0.251	0.392	0.306	0.688	0.284	0.164	2.258	7		
	Core&Peel	210	0.181	0.266	0.198	0.647	0.499	0.568	0.457	0.218	0.296	0.276	0.198	0.231	0.412	0.249	0.174	1.509	10		
	IMHRC	118	0.322	0.584	0.549	0.605	0.851	0.718	0.907	0.437	0.590	0.839	0.338	0.482	0.804	0.731	0.671	2.762	5		
	PC2P	251	0.568	0.863	0.671	0.679	0.899	0.781	0.717	0.713	0.715	0.665	0.570	0.614	1.182	0.760	0.761	3.600	2		

Data-set	Method	# Clusters	MMR	FRM	SEP	PPV	SN	ACC	Prec	Rec	F-measure	CDAP_prec	CDAP_rec	CDAP_fmeas	CDAP_F_MMR	Modularity	Overlapped Modularity	Composite Score (CS) MMR + FRM + SEP + ACC + F-measure	CS Ranking	Deviation of PC2P to Max in CS	Deviation of second best in CS compared to PC2P (if PC2P is best)
Gavin	MCL	128	0.312	0.570	0.568	0.509	0.914	0.682	0.797	0.433	0.561	0.695	0.306	0.425	0.736	0.856	0.624	2.693	2	0.00%	12.00%
	MCODE	87	0.209	0.333	0.490	0.562	0.654	0.606	0.770	0.265	0.394	0.701	0.210	0.323	0.532	0.651	0.538	2.033	8		
	CFinder	103	0.245	0.443	0.465	0.485	0.816	0.629	0.806	0.337	0.475	0.718	0.254	0.376	0.620	0.796	0.605	2.258	6		
	AP	301	0.264	0.478	0.289	0.366	0.719	0.513	0.292	0.357	0.322	0.219	0.227	0.223	0.487	0.383	0.290	1.866	9		
	CMC	138	0.239	0.399	0.394	0.649	0.568	0.607	0.717	0.337	0.458	0.522	0.247	0.336	0.575	0.429	0.334	2.097	7		
	ClusterOne	140	0.301	0.564	0.494	0.574	0.801	0.678	0.757	0.426	0.545	0.621	0.299	0.404	0.705	0.726	0.513	2.582	3		
	ProRank+	395	0.363	0.553	0.265	0.523	0.808	0.650	0.770	0.457	0.574	0.273	0.371	0.315	0.678	0.429	0.173	2.405	5		
	Core&Peel	176	0.179	0.282	0.248	0.682	0.484	0.575	0.631	0.230	0.337	0.330	0.199	0.248	0.427	0.332	0.173	1.621	10		
	IMHRC	127	0.269	0.557	0.485	0.538	0.843	0.673	0.669	0.357	0.466	0.575	0.251	0.349	0.618	0.759	0.541	2.450	4		
	PC2P	203	0.417	0.722	0.578	0.622	0.841	0.723	0.685	0.567	0.620	0.571	0.399	0.470	0.887	0.791	0.541	3.060	1		

Data-set	Method	# Clusters	MMR	FRM	SEP	PPV	SN	ACC	Prec	Rec	F-measure	CDAP_prec	CDAP_rec	CDAP_fmeas	CDAP_F_MMR	Modularity	Overlapped Modularity	Composite Score (CS) MMR + FRM + SEP + ACC + F-measure	CS Ranking	Deviation of PC2P to Max in CS	Deviation of second best in CS compared to PC2P (if PC2P is best)
KroganCore	MCL	176	0.371	0.569	0.585	0.531	0.882	0.685	0.795	0.454	0.578	0.744	0.379	0.502	0.873	0.714	0.480	2.789	2	0.00%	17.91%
	MCODE	65	0.117	0.191	0.366	0.581	0.367	0.462	0.677	0.136	0.226	0.569	0.107	0.180	0.297	0.388	0.376	1.362	8		
	CFinder	87	0.176	0.289	0.388	0.494	0.643	0.564	0.828	0.228	0.358	0.759	0.191	0.305	0.481	0.623	0.462	1.774	7		
	AP	244	0.279	0.462	0.351	0.345	0.711	0.495	0.451	0.350	0.394	0.328	0.231	0.271	0.550	0.417	0.341	1.982	5		
	CMC	102	0.121	0.211	0.267	0.659	0.312	0.454	0.549	0.150	0.236	0.412	0.121	0.188	0.308	0.240	0.107	1.289	9		
	ClusterOne	189	0.315	0.540	0.470	0.619	0.748	0.680	0.762	0.425	0.546	0.556	0.303	0.393	0.708	0.594	0.321	2.551	3		
	ProRank+	340	0.246	0.350	0.206	0.512	0.661	0.582	0.741	0.301	0.428	0.262	0.257	0.259	0.505	0.271	0.136	1.811	6		
	Core&Peel	144	0.085	0.150	0.143	0.652	0.252	0.405	0.472	0.101	0.167	0.201	0.084	0.118	0.204	0.176	0.070	0.950	10		
	IMHRC	161	0.263	0.517	0.457	0.550	0.791	0.660	0.677	0.321	0.435	0.528	0.246	0.335	0.598	0.611	0.343	2.332	4		
	PC2P	338	0.556	0.815	0.618	0.697	0.788	0.741	0.642	0.694	0.667	0.530	0.517	0.523	1.079	0.643	0.402	3.397	1		

Data-set	Method	# Clusters	MMR	FRM	SEP	PPV	SN	ACC	Prec	Rec	F-measure	CDAP_prec	CDAP_rec	CDAP_fmeas	CDAP_F_MMR	Modularity	Overlapped Modularity	Composite Score (CS) MMR + FRM + SEP + ACC + F-measure	CS Ranking	Deviation of PC2P to Max in CS	Deviation of second best in CS compared to PC2P (if PC2P is best)
KroganExt	MCL	236	0.362	0.590	0.555	0.582	0.774	0.671	0.674	0.466	0.551	0.568	0.359	0.440	0.802	0.517	0.138	2.729	2	0.00%	12.54%
	MCODE	54	0.089	0.145	0.334	0.491	0.353	0.417	0.648	0.097	0.168	0.593	0.086	0.150	0.239	0.293	0.100	1.152	9		
	CFinder	69	0.119	0.188	0.299	0.302	0.681	0.453	0.739	0.150	0.250	0.681	0.126	0.213	0.331	0.290	0.217	1.308	8		
	AP	320	0.275	0.483	0.298	0.335	0.593	0.446	0.384	0.330	0.355	0.203	0.174	0.188	0.463	0.237	0.090	1.856	5		
	CMC	125	0.135	0.231	0.280	0.616	0.351	0.465	0.624	0.182	0.282	0.440	0.147	0.221	0.355	0.194	-0.013	1.393	7		
	ClusterOne	144	0.222	0.378	0.419	0.610	0.591	0.600	0.792	0.311	0.447	0.646	0.249	0.360	0.582	0.380	0.193	2.067	3		
	ProRank+	206	0.173	0.273	0.239	0.570	0.550	0.560	0.680	0.217	0.329	0.330	0.182	0.235	0.408	0.202	0.112	1.574	6		
	Core&Peel	185	0.097	0.164	0.144	0.589	0.290	0.413	0.535	0.123	0.200	0.222	0.110	0.147	0.244	0.128	0.009	1.018	10		
	IMHRC	192	0.229	0.491	0.365	0.472	0.802	0.615	0.490	0.239	0.321	0.359	0.185	0.244	0.473	0.471	0.137	2.020	4		
	PC2P	422	0.504	0.775	0.560	0.670	0.717	0.693	0.536	0.651	0.588	0.384	0.434	0.408	0.912	0.482	0.073	3.120	1		

Supplementary Table 3. Comparative analysis of eight algorithms for prediction of protein complexes with respect to nine performance measures as well as a composite score that combines five of them. Shown is also the modularity of the resulting clusterings.

Comparative analysis

Yeast - SGD

Data-set	Method	# Clusters	MMR	FRM	SEP	PPV	SN	ACC	Prec	Rec	F-measure	CDAP_prec	CDAP_rec	CDAP_fmeas	CDAP_F_MMR	Modularity	Overlapped Modularity	Composite Score (CS) MMR + FRM + SEP + ACC + F-measure	CS Ranking	Deviation of PC2P to Max in CS	Deviation of second best in CS compared to PC2P (if PC2P is best)
Collins	MCL	156	0.508	0.791	0.671	0.566	0.863	0.699	0.859	0.643	0.735	0.821	0.514	0.632	1.140	0.891	0.804	3.404	2	0.00%	0.16%
	MCODE	85	0.262	0.438	0.503	0.541	0.630	0.584	0.800	0.333	0.471	0.800	0.273	0.407	0.670	0.713	0.700	2.258	8		
	CFinder	83	0.272	0.474	0.503	0.484	0.758	0.606	0.855	0.337	0.484	0.843	0.281	0.422	0.693	0.831	0.796	2.339	7		
	AP	272	0.507	0.787	0.425	0.452	0.698	0.562	0.515	0.635	0.568	0.445	0.486	0.464	0.972	0.462	0.692	2.850	3		
	CMC	74	0.203	0.349	0.370	0.525	0.534	0.529	0.851	0.285	0.427	0.757	0.225	0.347	0.549	0.521	0.557	1.879	9		
	ClusterOne	104	0.338	0.570	0.533	0.551	0.759	0.647	0.885	0.458	0.603	0.865	0.361	0.510	0.848	0.807	0.727	2.692	4		
	ProRank+	314	0.407	0.562	0.275	0.446	0.761	0.583	0.803	0.462	0.586	0.322	0.406	0.359	0.765	0.474	0.226	2.413	6		
	Core&Peel	106	0.181	0.305	0.243	0.496	0.502	0.499	0.736	0.241	0.363	0.472	0.201	0.282	0.463	0.405	0.319	1.592	10		
	IMHRC	103	0.335	0.566	0.527	0.548	0.766	0.648	0.883	0.438	0.585	0.854	0.353	0.500	0.835	0.784	0.718	2.661	5		
	PC2P	182	0.525	0.827	0.644	0.580	0.842	0.699	0.758	0.675	0.714	0.725	0.530	0.613	1.137	0.873	0.805	3.409	1		

Data-set	Method	# Clusters	MMR	FRM	SEP	PPV	SN	ACC	Prec	Rec	F-measure	CDAP_prec	CDAP_rec	CDAP_fmeas	CDAP_F_MMR	Modularity	Overlapped Modularity	Composite Score (CS) MMR + FRM + SEP + ACC + F-measure	CS Ranking	Deviation of PC2P to Max in CS	Deviation of second best in CS compared to PC2P (if PC2P is best)
Gavin	MCL	97	0.291	0.510	0.537	0.430	0.895	0.621	0.825	0.382	0.523	0.753	0.291	0.420	0.711	0.827	0.709	2.481	3	0.00%	17.18%
	MCODE	77	0.220	0.394	0.464	0.504	0.648	0.572	0.779	0.267	0.398	0.714	0.219	0.335	0.555	0.647	0.576	2.047	7		
	CFinder	83	0.244	0.470	0.462	0.437	0.808	0.594	0.795	0.303	0.439	0.771	0.255	0.383	0.627	0.793	0.668	2.209	6		
	AP	226	0.277	0.498	0.297	0.349	0.636	0.471	0.332	0.339	0.335	0.261	0.235	0.247	0.524	0.450	0.334	1.878	9		
	CMC	107	0.228	0.398	0.374	0.535	0.561	0.548	0.673	0.303	0.418	0.533	0.227	0.318	0.546	0.451	0.388	1.966	8		
	ClusterOne	109	0.293	0.598	0.492	0.511	0.809	0.643	0.780	0.406	0.534	0.716	0.311	0.433	0.726	0.778	0.589	2.560	2		
	ProRank+	290	0.352	0.554	0.273	0.455	0.797	0.602	0.759	0.406	0.529	0.310	0.359	0.333	0.685	0.480	0.211	2.310	5		
	Core&Peel	132	0.187	0.319	0.245	0.510	0.493	0.501	0.621	0.227	0.333	0.364	0.191	0.251	0.437	0.370	0.237	1.585	10		
	IMHRC	104	0.273	0.566	0.474	0.483	0.809	0.625	0.769	0.382	0.511	0.673	0.279	0.394	0.668	0.778	0.600	2.449	4		
	PC2P	159	0.421	0.753	0.578	0.567	0.822	0.683	0.761	0.578	0.657	0.654	0.414	0.507	0.928	0.810	0.630	3.091	1		

Data-set	Method	# Clusters	MMR	FRM	SEP	PPV	SN	ACC	Prec	Rec	F-measure	CDAP_prec	CDAP_rec	CDAP_fmeas	CDAP_F_MMR	Modularity	Overlapped Modularity	Composite Score (CS) MMR + FRM + SEP + ACC + F-measure	CS Ranking	Deviation of PC2P to Max in CS	Deviation of second best in CS compared to PC2P (if PC2P is best)
KroganCore	MCL	140	0.374	0.565	0.546	0.460	0.804	0.608	0.800	0.471	0.593	0.736	0.373	0.495	0.869	0.785	0.536	2.686	2	0.00%	15.72%
	MCODE	54	0.126	0.239	0.367	0.466	0.387	0.425	0.685	0.156	0.254	0.611	0.120	0.200	0.326	0.425	0.417	1.411	8		
	CFinder	73	0.193	0.333	0.399	0.422	0.632	0.516	0.877	0.257	0.398	0.795	0.210	0.332	0.526	0.635	0.493	1.839	7		
	AP	208	0.315	0.507	0.330	0.290	0.704	0.452	0.433	0.359	0.392	0.370	0.279	0.318	0.633	0.437	0.342	1.997	5		
	CMC	81	0.130	0.254	0.274	0.551	0.335	0.430	0.605	0.167	0.261	0.506	0.149	0.230	0.359	0.271	0.185	1.348	9		
	ClusterOne	137	0.324	0.507	0.475	0.516	0.703	0.602	0.796	0.428	0.556	0.664	0.330	0.441	0.765	0.622	0.374	2.465	3		
	ProRank+	297	0.278	0.399	0.210	0.423	0.639	0.520	0.717	0.341	0.462	0.269	0.290	0.279	0.557	0.283	0.140	1.867	6		
	Core&Peel	130	0.104	0.192	0.140	0.500	0.289	0.380	0.477	0.127	0.200	0.231	0.109	0.148	0.251	0.192	0.085	1.016	10		
	IMHRC	122	0.282	0.518	0.456	0.500	0.734	0.606	0.754	0.355	0.483	0.623	0.275	0.382	0.664	0.687	0.390	2.344	4		
	PC2P	256	0.516	0.793	0.564	0.583	0.738	0.656	0.637	0.681	0.658	0.543	0.504	0.523	1.039	0.698	0.469	3.187	1		

Data-set	Method	# Clusters	MMR	FRM	SEP	PPV	SN	ACC	Prec	Rec	F-measure	CDAP_prec	CDAP_rec	CDAP_fmeas	CDAP_F_MMR	Modularity	Overlapped Modularity	Composite Score (CS) MMR + FRM + SEP + ACC + F-measure	CS Ranking	Deviation of PC2P to Max in CS	Deviation of second best in CS compared to PC2P (if PC2P is best)
KroganExt	MCL	71	0.170	0.243	0.357	0.189	0.906	0.414	0.704	0.189	0.298	0.662	0.159	0.256	0.426	0.194	0.475	1.483	6	0.00%	25.83%
	MCODE	39	0.080	0.122	0.310	0.338	0.372	0.355	0.692	0.098	0.172	0.667	0.088	0.155	0.235	0.318	0.227	1.038	10		
	CFinder	57	0.127	0.209	0.303	0.253	0.639	0.402	0.789	0.162	0.269	0.684	0.132	0.221	0.348	0.276	0.271	1.311	8		
	AP	235	0.239	0.422	0.279	0.299	0.543	0.403	0.340	0.287	0.312	0.221	0.176	0.196	0.435	0.286	0.109	1.655	4		
	CMC	97	0.151	0.267	0.288	0.519	0.359	0.432	0.629	0.203	0.307	0.485	0.159	0.239	0.390	0.214	0.055	1.445	7		
	ClusterOne	116	0.250	0.392	0.433	0.529	0.569	0.549	0.862	0.328	0.475	0.724	0.284	0.408	0.658	0.402	0.264	2.099	2		
	ProRank+	185	0.207	0.318	0.249	0.453	0.530	0.490	0.692	0.257	0.375	0.351	0.220	0.270	0.477	0.212	0.124	1.638	5		
	Core&Peel	149	0.114	0.199	0.153	0.495	0.310	0.392	0.510	0.142	0.222	0.242	0.122	0.162	0.276	0.149	0.038	1.080	9		
	IMHRC	149	0.240	0.459	0.360	0.420	0.704	0.543	0.570	0.291	0.385	0.443	0.223	0.297	0.537	0.503	0.177	1.988	3		
	PC2P	324	0.469	0.716	0.502	0.565	0.663	0.612	0.485	0.588	0.531	0.386	0.422	0.403	0.872	0.533	0.201	2.830	1		

Supplementary Table 3. Comparative analysis of eight algorithms for prediction of protein complexes with respect to nine performance measures as well as a composite score that combines five of them. Shown is also the modularity of the resulting clusterings.

Comparative analysis

Human - Corum

Data-set	Method	# Clusters	MMR	FRM	SEP	PPV	SN	ACC	Prec	Rec	F-measure	CDAP_prec	CDAP_rec	CDAP_fmeas	CDAP_F_MMR	Modularity	Overlapped Modularity	Composite Score (CS) MMR + FRM + SEP + ACC + F-measure	CS Ranking	Deviation of PC2P to Max in CS	Deviation of second best in CS compared to PC2P (if PC2P is best)
STRING	MCL	384	0.089	0.606	0.237	0.139	0.754	0.323	0.802	0.404	0.537	0.664	0.085	0.150	0.239	0.721	0.655	1.792	2	0.00%	0.15%
	MCODE	114	0.026	0.198	0.156	0.145	0.378	0.234	0.763	0.119	0.206	0.728	0.028	0.053	0.079	0.523	0.495	0.820	8		
	CFinder	119	0.030	0.263	0.159	0.125	0.569	0.266	0.866	0.146	0.249	0.807	0.032	0.061	0.091	0.693	0.621	0.967	7		
	AP	501	0.078	0.512	0.160	0.098	0.738	0.268	0.537	0.342	0.418	0.419	0.070	0.119	0.197	0.316	0.558	1.435	3		
	CMC	132	0.023	0.132	0.094	0.148	0.283	0.205	0.636	0.089	0.155	0.523	0.023	0.044	0.067	0.330	0.246	0.608	9		
	ClusterOne	243	0.053	0.419	0.183	0.128	0.652	0.289	0.794	0.234	0.362	0.646	0.052	0.096	0.149	0.651	0.491	1.305	4		
	ProRank+	587	0.083	0.349	0.080	0.140	0.579	0.285	0.714	0.235	0.354	0.443	0.086	0.144	0.227	0.275	0.145	1.151	6		
	Core&Peel	206	0.025	0.091	0.049	0.143	0.241	0.186	0.515	0.059	0.106	0.374	0.026	0.048	0.073	0.191	0.124	0.458	10		
	IMHRC	213	0.047	0.398	0.188	0.127	0.732	0.304	0.756	0.196	0.311	0.648	0.046	0.085	0.133	0.679	0.517	1.248	5		
	PC2P	462	0.094	0.616	0.227	0.141	0.755	0.326	0.723	0.421	0.532	0.546	0.084	0.145	0.239	0.721	0.651	1.795	1		

Data-set	Method	# Clusters	MMR	FRM	SEP	PPV	SN	ACC	Prec	Rec	F-measure	CDAP_prec	CDAP_rec	CDAP_fmeas	CDAP_F_MMR	Modularity	Overlapped Modularity	Composite Score (CS) MMR + FRM + SEP + ACC + F-measure	CS Ranking	Deviation of PC2P to Max in CS	Deviation of second best in CS compared to PC2P (if PC2P is best)
PIPS	MCL	73	0.018	0.119	0.127	0.043	0.906	0.196	0.740	0.099	0.174	0.452	0.014	0.027	0.044	0.298	0.593	0.634	4	0.00%	24.35%
	MCODE	32	0.006	0.060	0.106	0.069	0.292	0.141	0.406	0.015	0.029	0.281	0.004	0.007	0.013	0.221	0.192	0.341	9		
	CFinder	36	0.008	0.061	0.090	0.043	0.621	0.163	0.556	0.023	0.044	0.417	0.006	0.012	0.020	0.391	0.435	0.366	8		
	AP	222	0.051	0.371	0.142	0.064	0.687	0.210	0.635	0.267	0.376	0.392	0.036	0.066	0.117	0.293	0.356	1.150	2		
	CMC	68	0.009	0.103	0.077	0.057	0.359	0.143	0.338	0.065	0.108	0.235	0.007	0.013	0.022	0.132	0.024	0.440	7		
	ClusterOne	83	0.015	0.131	0.124	0.075	0.477	0.189	0.386	0.048	0.085	0.301	0.010	0.020	0.035	0.394	0.297	0.544	6		
	ProRank+	212	0.030	0.234	0.059	0.054	0.569	0.176	0.396	0.096	0.154	0.250	0.022	0.040	0.070	0.128	0.113	0.653	3		
	Core&Peel	123	0.010	0.063	0.046	0.058	0.300	0.132	0.203	0.026	0.047	0.138	0.007	0.013	0.023	0.088	0.031	0.298	10		
	IMHRC	99	0.017	0.110	0.126	0.062	0.699	0.208	0.374	0.055	0.097	0.303	0.012	0.024	0.041	0.470	0.278	0.557	5		
	PC2P	302	0.078	0.495	0.218	0.098	0.713	0.264	0.689	0.351	0.465	0.454	0.057	0.101	0.178	0.494	0.366	1.520	1		

Supplementary Table 4. Resulst of the post-hoc Tukey test comparing the means of GO semantic similarity over the clusters for biological process (BP), molecular function (MF), and cellular compartment (CC). Results are show for min and mean analyses for four data sets of yeast and two data sets of human.

Tukey test			Mean									Min								
			BP			MF			CC			BP			MF			CC		
			diff	p.adj	Interpretation	diff	p.adj	Interpretation	diff	p.adj	Interpretation	diff	p.adj	Interpretation	diff	p.adj	Interpretation	diff	p.adj	Interpretation
		MCODE-MCL	0.01329	0.99861	-	-0.00767	0.99999	-	-0.00694	0.99881	-	0.00002	1.00000	-	-0.05403	0.75399	-	-0.02426	0.97391	-
		CFinder-MCL	-0.02877	0.76888	-	-0.03909	0.46987	-	-0.01350	0.87609	-	-0.06270	0.59545	-	-0.10669	0.01535	CFinder < MCL	-0.04494	0.43829	-
		AP-MCL	-0.00760	0.99989	-	-0.00698	0.99997	-	-0.00723	0.98932	-	-0.00325	1.00000	-	0.00283	1.00000	-	-0.00995	0.99981	-
		CMC-MCL	-0.03229	0.72577	-	-0.05980	0.06257	-	-0.03402	0.01225	CMC < MCL	-0.12992	0.00478	CMC < MCL	-0.16939	0.00001	CMC < MCL	-0.10811	0.00003	CMC < MCL
		ClusterOne-MCL	-0.06585	0.00006	ClusterOne < MCL	-0.06732	0.00023	ClusterOne < MCL	-0.03243	0.00031	ClusterOne < MCL	-0.13829	0.00000	ClusterOne < MCL	-0.14870	0.00000	ClusterOne < MCL	-0.08950	0.00000	ClusterOne < MCL
		ProRank+-MCL	-0.01977	0.66250	-	-0.07593	0.00000	ProRank+ < MCL	-0.03410	0.00000	ProRank+ < MCL	-0.13979	0.00000	ProRank+ < MCL	-0.21743	0.00000	ProRank+ < MCL	-0.12199	0.00000	ProRank+ < MCL
		Core&Peel-MCL	0.01836	0.88840	-	-0.01290	0.99358	-	0.00226	1.00000	-	-0.05614	0.30591	-	-0.14380	0.00000	Core&Peel < MCL	-0.03454	0.37591	-
		IMHRC-MCL	-0.07070	0.00002	IMHRC < MCL	-0.07551	0.00003	IMHRC < MCL	-0.03950	0.00000	IMHRC < MCL	-0.15169	0.00000	IMHRC < MCL	-0.16171	0.00000	IMHRC < MCL	-0.10980	0.00000	IMHRC < MCL
		PC2P-MCL	0.00660	0.99994	-	0.00445	1.00000	-	0.00485	0.99918	-	0.00849	1.00000	-	0.00645	1.00000	-	0.01090	0.99932	-
		CFinder-MCODE	-0.04207	0.53245	-	-0.03142	0.91528	-	-0.00656	0.99984	-	-0.06271	0.82593	-	-0.05266	0.91828	-	-0.02068	0.99793	-
		AP-MCODE	-0.02090	0.97187	-	0.00069	1.00000	-	-0.00030	1.00000	-	-0.00327	1.00000	-	0.05686	0.74396	-	0.01430	0.99965	-
		CMC-MCODE	-0.04559	0.49079	-	-0.05213	0.41407	-	-0.02708	0.32411	-	-0.12994	0.04092	CMC < MCODE	-0.11536	0.08806	-	-0.08385	0.04094	CMC < MCODE
		ClusterOne-MCODE	-0.07914	0.00038	ClusterOne < MCODE	-0.05965	0.06391	-	-0.02549	0.17653	-	-0.13831	0.00176	ClusterOne < MCODE	-0.09467	0.10551	-	-0.06524	0.08204	-
		ProRank+-MCODE	-0.03306	0.50150	-	-0.06826	0.00208	ProRank+ < MCODE	-0.02716	0.03311	ProRank+ < MCODE	-0.13981	0.00009	ProRank+ < MCODE	-0.16340	0.00000	ProRank+ < MCODE	-0.09773	0.00001	ProRank+ < MCODE
		Core&Peel-MCODE	0.00507	1.00000	-	-0.00523	1.00000	-	0.00919	0.99029	-	-0.05616	0.75510	-	-0.08977	0.09922	-	-0.01028	0.99997	-
		IMHRC-MCODE	-0.08399	0.00015	IMHRC < MCODE	-0.06784	0.01966	IMHRC < MCODE	-0.03257	0.02437	IMHRC < MCODE	-0.15171	0.00041	IMHRC < MCODE	-0.10768	0.03814	IMHRC < MCODE	-0.08554	0.00435	IMHRC < MCODE
		PC2P-MCODE	-0.00669	1.00000	-	0.01212	0.99968	-	0.01179	0.94865	-	0.00847	1.00000	-	0.06048	0.62181	-	0.03516	0.78801	-
		AP-CFinder	0.02117	0.96752	-	0.03212	0.78598	-	0.00626	0.99960	-	0.05945	0.72167	-	0.10953	0.01876	AP > CFinder	0.03498	0.81734	-
		CMC-CFinder	-0.00352	1.00000	-	-0.02070	0.99638	-	-0.02052	0.71464	-	-0.06722	0.80566	-	-0.06269	0.83686	-	-0.06317	0.29675	-
		ClusterOne-CFinder	-0.03707	0.52625	-	-0.02823	0.90339	-	-0.01893	0.58634	-	-0.07560	0.41668	-	-0.04201	0.95416	-	-0.04456	0.55387	-
		ProRank+-CFinder	0.00900	0.99989	-	-0.03684	0.44708	-	-0.02060	0.25204	-	-0.07709	0.19268	-	-0.11073	0.00325	ProRank+ < CFinder	-0.07705	0.00165	ProRank+ < CFinder
		Core&Peel-CFinder	0.04713	0.12257	-	0.02619	0.90998	-	0.01575	0.74376	-	0.00655	1.00000	-	-0.03710	0.96919	-	0.01040	0.99996	-
	Collin	IMHRC-CFinder	-0.04192	0.36110	-	-0.03642	0.68816	-	-0.02601	0.16092	-	-0.08899	0.20789	-	-0.05502	0.80864	-	-0.06486	0.09012	-
		PC2P-CFinder	0.03537	0.50530	-	0.04354	0.31849	-	0.01835	0.54308	-	0.07118	0.41555	-	0.11314	0.00784	PC2P > CFinder	0.05584	0.15546	-
		CMC-AP	-0.02469	0.94500	-	-0.05282	0.20367	-	-0.02678	0.16320	-	-0.12667	0.01136	CMC < AP	-0.17222	0.00002	CMC < AP	-0.09815	0.00062	CMC < AP
		ClusterOne-AP	-0.05824	0.00229	ClusterOne < AP	-0.06034	0.00488	ClusterOne < AP	-0.02519	0.03526	ClusterOne < AP	-0.13504	0.00004	ClusterOne < AP	-0.15153	0.00000	ClusterOne < AP	-0.07955	0.00031	ClusterOne < AP
		ProRank+-AP	-0.01217	0.98822	-	-0.06896	0.00000	ProRank+ < AP	-0.02686	0.00047	ProRank+ < AP	-0.13654	0.00000	ProRank+ < AP	-0.22026	0.00000	ProRank+ < AP	-0.11203	0.00000	ProRank+ < AP
		Core&Peel-AP	0.02596	0.61390	-	-0.00592	0.99999	-	0.00949	0.93903	-	-0.05289	0.50986	-	-0.14663	0.00000	Core&Peel < AP	-0.02459	0.88001	-
		IMHRC-AP	-0.06309	0.00082	IMHRC < AP	-0.06854	0.00083	IMHRC < AP	-0.03227	0.00164	IMHRC < AP	-0.14844	0.00001	IMHRC < AP	-0.16454	0.00000	IMHRC < AP	-0.09984	0.00000	IMHRC < AP
		PC2P-AP	0.01420	0.98660	-	0.01143	0.99861	-	0.01209	0.77943	-	0.01174	0.99998	-	0.00362	1.00000	-	0.02086	0.95484	-
		ClusterOne-CMC	-0.03355	0.74923	-	-0.00752	1.00000	-	0.00159	1.00000	-	-0.00838	1.00000	-	0.02069	0.99987	-	0.01861	0.99852	-
		ProRank+-CMC	0.01252	0.99916	-	-0.01614	0.99683	-	-0.00008	1.00000	-	-0.00987	1.00000	-	-0.04804	0.86346	-	-0.01388	0.99963	-
		Core&Peel-CMC	0.05065	0.12596	-	0.04690	0.31888	-	0.03627	0.00544	Core&Peel > CMC	0.07378	0.47469	-	0.02559	0.99884	-	0.07357	0.02725	Core&Peel > CMC
		IMHRC-CMC	-0.03840	0.59196	-	-0.01572	0.99910	-	-0.00549	0.99994	-	-0.02177	0.99986	-	0.00768	1.00000	-	-0.00169	1.00000	-
		PC2P-CMC	0.03889	0.47601	-	0.06425	0.03304	PC2P > CMC	0.03887	0.00187	PC2P > CMC	0.13841	0.00193	PC2P > CMC	0.17584	0.00000	PC2P > CMC	0.11901	0.00000	PC2P > CMC
		ProRank+-ClusterOne	0.04608	0.00566	ProRank+ > ClusterOne	-0.00861	0.99972	-	-0.00167	1.00000	-	-0.00150	1.00000	-	-0.06873	0.06194	-	-0.03249	0.46377	-
		Core&Peel-ClusterOne	0.08421	0.00000	Core&Peel > ClusterOne	0.05442	0.00982	Core&Peel > ClusterOne	0.03468	0.00009	Core&Peel > ClusterOne	0.08215	0.05052	-	0.00490	1.00000	-	0.05496	0.03534	Core&Peel > ClusterOne
		IMHRC-ClusterOne	-0.00485	1.00000	-	-0.00819	0.99997	-	-0.00708	0.99725	-	-0.01339	0.99999	-	-0.01301	0.99998	-	-0.02029	0.98606	-
		PC2P-ClusterOne	0.07245	0.00001	PC2P > ClusterOne	0.07177	0.00007	PC2P > ClusterOne	0.03728	0.00002	PC2P > ClusterOne	0.14678	0.00000	PC2P > ClusterOne	0.15515	0.00000	PC2P > ClusterOne	0.10041	0.00000	PC2P > ClusterOne
		Core&Peel-ProRank+	0.03813	0.01076	Core&Peel > ProRank+	0.06303	0.00000	Core&Peel > ProRank+	0.03635	0.00000	Core&Peel > ProRank+	0.08365	0.00116	Core&Peel > ProRank+	0.07363	0.00526	Core&Peel > ProRank+	0.08745	0.00000	Core&Peel > ProRank+
		IMHRC-ProRank+	-0.05093	0.00188	IMHRC < ProRank+	0.00042	1.00000	-	-0.00541	0.99837	-	-0.01190	0.99997	-	0.05572	0.30506	-	0.01219	0.99862	-
		PC2P-ProRank+	0.02637	0.26749	-	0.08038	0.00000	PC2P > ProRank+	0.03895	0.00000	PC2P > ProRank+	0.14828	0.00000	PC2P > ProRank+	0.22388	0.00000	PC2P > ProRank+	0.13289	0.00000	PC2P > ProRank+
		IMHRC-Core&Peel	-0.08906	0.00000	IMHRC < Core&Peel	-0.06261	0.00167	IMHRC < Core&Peel	-0.04176	0.00000	IMHRC < Core&Peel	-0.09555	0.01226	IMHRC < Core&Peel	-0.01791	0.99953	-	-0.07526	0.00051	IMHRC < Core&Peel
		PC2P-Core&Peel	-0.01176	0.99453	-	0.01735	0.95484	-	0.00260	1.00000	-	0.06463	0.14884	-	0.15025	0.00000	PC2P > Core&Peel	0.04545	0.07793	-
		PC2P-IMHRC	0.07730	0.00000	PC2P > IMHRC	0.07996	0.00001	PC2P > IMHRC	0.04435	0.00000	PC2P > IMHRC	0.16018	0.00000	PC2P > IMHRC	0.16816	0.00000	PC2P > IMHRC	0.12070	0.00000	PC2P > IMHRC
		MCODE-MCL	0.01619	0.99528	-	-0.02121	0.94469	-	0.00928	0.99554	-	0.02819	0.99234	-	-0.06180	0.21395	-	0.01065	0.99996	-
		CFinder-MCL	-0.05248	0.01642	CFinder < MCL	-0.03947	0.11110	-	-0.02359	0.16096	-	-0.07203	0.09159	-	-0.05731	0.13727	-	-0.05036	0.12871	-
		AP-MCL	-0.04967	0.03323	AP < MCL	-0.03469	0.26103	-	-0.02779	0.04572	AP < MCL	-0.04448	0.72826	-	-0.02742	0.94699	-	-0.05007	0.14117	-
		CMC-MCL	0.03892	0.17016	-	-0.00791	0.99986	-	0.00963	0.97788	-	0.03140	0.94415	-	-0.07144	0.01076	CMC < MCL	0.00130	1.00000	-
		ClusterOne-MCL	-0.04626	0.02087	ClusterOne < MCL	-0.05338	0.00064	ClusterOne < MCL	-0.02848	0.00937	ClusterOne < MCL	-0.07185	0.03560	ClusterOne < MCL	-0.09195	0.00003	ClusterOne < MCL	-0.07583	0.00010	ClusterOne < MCL
		ProRank+-MCL	0.03864	0.02150	ProRank+ > MCL	-0.02667	0.22476	-	0.00687	0.98855	-	-0.02006	0.98554	-	-0.11072	0.00000	ProRank+ < MCL	-0.04184	0.05822	-
		Core&Peel-MCL	0.09012	0.00000	Core&Peel > MCL	0.00347	1.00000	-	0.03624	0.00002	Core&Peel > MCL	0.09843	0.00005	Core&Peel > MCL	-0.07359	0.00063	Core&Peel < MCL	0.05214	0.01536	Core&Peel > MCL
		IMHRC-MCL	0.02996	0.59903	-	-0.01334	0.99374	-	0.00686	0.99868	-	0.01799	0.99927	-	-0.05845	0.12095	-	-0.01203	0.99965	-
		PC2P-MCL	-0.00029	1.00000	-	-0.00811	0.99959	-	0.00399	0.99995	-	0.00307	1.00000	-	-0.01800	0.99130	-	0.00684	0.99999	-
		CFinder-MCODE	-0.06868	0.01412	CFinder < MCODE	-0.01826	0.99037	-	-0.03287	0.09367	-	-0.10022	0.04791	CFinder < MCODE	0.00449	1.00000	-	-0.06101	0.19638	-
		AP-MCODE	-0.06587	0.02439	AP < MCODE	-0.01348	0.99908	-	-0.03706	0.03223	AP < MCODE	-0.07267	0.39176	-	0.03438	0.95538	-	-0.06071	0.20736	-
		CMC-MCODE	0.02273	0.97184	-	0.01330	0.99897	-	0.00035	1.00000	-	0.00321	1.00000	-	-0.00964	1.00000	-	-0.00935	0.99999	-
		ClusterOne-MCODE	-0.06245	0.02130	ClusterOne < MCODE	-0.03218	0.64743	-	-0.03776	0.01216	ClusterOne < MCODE	-0.10004	0.02608	ClusterOne < MCODE	-0.03015	0.97088	-	-0.08648	0.00276	ClusterOne < MCODE
		ProRank+-MCODE	0.02245	0.94180	-	-0.00547	1.00000	-	-0.00241	1.00000	-	-0.04825	0.74890	-	-0.04892	0.49413	-	-0.05249	0.19708	-
		Core&Peel-MCODE	0.07393	0.00094	Core&Peel > MCODE	0.02468	0.87289	-	0.02696	0.18157	-	0.07024	0.28321	-	-0.01179	0.99997	-	0.04149	0.60072	-
		IMHRC-MCODE	0.01376	0.99945	-	0.00787	0.99999	-	-0.00242	1.00000	-	-0.01020	1.00000	-	0.00335	1.00000	-	-0.02267	0.99323	-
		PC2P-MCODE	-0.01649	0.99564	-	0.01309	0.99855	-	-0.00529	0.99996	-	-0.02512	0.99741	-	0.04380	0.73748	-	-0.00380	1.00000	-
		AP-CFinder	0.00281	1.00000	-	0.00478	1.00000	-	-0.00420	0.99999	-	0.02755	0.99368	-	0.02989	0.96263	-	0.00030	1.00000	-
		CMC-CFinder	0.09140	0.00000	CMC > CFinder	0.03156	0.55841	-	0.03322	0.02122	CMC > CFinder	0.10343	0.00604	CMC > CFinder	-0.01412	0.99983	-	0.05166	0.22465	-
		ClusterOne-CFinder	0.00622	1.00000	-	-0.01392	0.99469	-	-0.00489	0.99995	-	0.00018	1.00000	-	-0.03464	0.85641	-	-0.02547	0.94506	-
		ProRank+-CFinder	0.09113	0.00000	ProRank+ > CFinder	0.01279	0.99312	-	0.03046	0.00773	ProRank+ > CFinder	0.05198	0.42402	-	-0.05341	0.15461	-	0.00852	0.99997	-
		Core&Peel-CFinder	0.14261	0.00000	Core&Peel > CFinder	0.04294	0.05960	-	0.05982	0.00000	Core&Peel > CFinder	0.17046	0.00000	Core&Peel > CFinder	-0.01628	0.99876	-	0.10250	0.00000	Core&Peel > CFinder
	Gavin	IMHRC-CFinder	0.08244	0.00008	IMHRC > CFinder	0.02613	0.82288	-	0.03045	0.06972	-	0.09002	0.04588	IMHRC > CFinder	-0.00114	1.00000	-	0.03833	0.69616	-
		PC2P-CFinder	0.05219	0.02713	PC2P > CFinder	0.03135	0.45394	-	0.02757	0.06522	-	0.07511	0.08875	-	0.03932	0.70396	-	0.05720	0.06287	-
		CMC-AP	0.08859	0.00001	CMC > AP	0.02678	0.77529	-	0.03742	0.00479	CMC > AP	0.07588	0.15017	-	-0.04401	0.66430	-	0.05137	0.23936	-
		ClusterOne-AP	0.00341	1.00000	-	-0.01870	0.96025	-	-0.00069	1.00000	-	-0.02737	0.98949	-	-0.06453	0.09765	-	-0.02576	0.94332	-
		ProRank+-AP	0.08832	0.00000	ProRank+ > AP	0.00801	0.99984	-	0.03466	0.00114	ProRank+ > AP	0.02443	0.98922	-	-0.08330	0.00091	ProRank+ < AP	0.00823	0.99998	-
		Core&Peel-AP	0.13980	0.00000	Core&Peel > AP	0.03816	0.15779	-	0.06402	0.00000	Core&Peel > AP	0.14291	0.00000	Core&Peel > AP	-0.04617	0.44450	-	0.10221	0.00000	Core&Peel > AP
		IMHRC-AP	0.07963	0.00021	IMHRC > AP	0.02135	0.94402	-	0.03465	0.01990	IMHRC > AP	0.06247	0.45354	-	-0.03103	0.95304	-	0.03804	0.71238	-
		PC2P-AP	0.04938	0.05116	-	0.02657	0.69919	-	0.03177	0.01588	PC2P > AP	0.04756	0.69263	-	0.00943	0.99999	-	0.05691	0.06988	-
		ClusterOne-CMC	-0.08518	0.00000	ClusterOne < CMC	-0.04548	0.04262	ClusterOne < CMC	-0.03811	0.00081	ClusterOne < CMC	-0.10325	0.00170	ClusterOne < CMC	-0.02051	0.99381	-	-0.07713	0.00118	ClusterOne < CMC
		ProRank+-CMC	-0.00028	1.00000	-	-0.01877	0.88815	-	-0.00276	1.00000	-	-0.05146	0.37150	-	-0.03929	0.51683	-	-0.04314	0.18754	-
		Core&Peel-CMC	0.05120	0.01536	Core&Peel > CMC	0.01138	0.99761	-	0.02660	0.04843	Core&Peel > CMC	0.06703	0.12570	-	-0.00215	1.00000	-	0.05084	0.09565	-
		IMHRC-CMC	-0.00896	0.99995	-	-0.00544	1.00000	-	-0.00277	1.00000	-	-0.01342	0.99998	-	0.01299	0.99992	-	-0.01333	0.99968	-
		PC2P-CMC	-0.03921	0.20832	-	-0.00021	1.00000	-	-0.00565	0.99973	-	-0.02833	0.97797	-	0.05344	0.21461	-	0.00554	1.00000	-
		ProRank+-ClusterOne	0.08490	0.00000	ProRank+ > ClusterOne	0.02671	0.37807	-	0.03535	0.00005	ProRank+ > ClusterOne	0.05179	0.25592	-	-0.01877	0.98582	-	0.03399	0.41269	-
		Core&Peel-ClusterOne	0.13638	0.00000	Core&Peel > ClusterOne	0.05686	0.00022	Core&Peel > ClusterOne	0.06471	0.00000	Core&Peel > ClusterOne	0.17028	0.00000	Core&Peel > ClusterOne	0.01836	0.99307	-	0.12797	0.00000	Core&Peel > ClusterOne
		IMHRC-ClusterOne	0.07621	0.00009	IMHRC > ClusterOne	0.04004	0.16114	-	0.03534	0.00517	IMHRC > ClusterOne	0.08984	0.02062	IMHRC > ClusterOne	0.03350	0.88067	-	0.06380	0.02868	IMHRC > ClusterOne
		PC2P-ClusterOne	0.04597	0.03711	PC2P > ClusterOne	0.04527	0.01655	PC2P > ClusterOne	0.03246	0.00270	PC2P > ClusterOne	0.07492	0.03730	PC2P > ClusterOne	0.07395	0.00506	PC2P > ClusterOne	0.08267	0.00004	PC2P > ClusterOne
		Core&Peel-ProRank+	0.05148	0.00030	Core&Peel > ProRank+	0.03015	0.11269	-	0.02937	0.00039	Core&Peel > ProRank+	0.11849	0.00000	Core&Peel > ProRank+	0.03713	0.34471	-	0.09398	0.00000	Core&Peel > ProRank+
		IMHRC-ProRank+	-0.00869	0.99984	-	0.01333	0.99084	-	-0.00001	1.00000	-	0.03804	0.82734	-	0.05227	0.17975	-	0.02981	0.76080	-
		PC2P-ProRank+	-0.03894	0.03989	PC2P < ProRank+	0.01856	0.80359	-	-0.00288	0.99999	-	0.02313	0.97536	-	0.09273	0.00000	PC2P > ProRank+	0.04868	0.02416	PC2P > ProRank+
		IMHRC-Core&Peel	-0.06017	0.00282	IMHRC < Core&Peel	-0.01681	0.97021	-	-0.02937	0.02666	IMHRC < Core&Peel	-0.08045	0.03702	IMHRC < Core&Peel	0.01514	0.99931	-	-0.06417	0.01384	IMHRC < Core&Peel
		PC2P-Core&Peel	-0.09042	0.00000	PC2P < Core&Peel	-0.01159	0.99398	-	-0.03225	0.00083	PC2P < Core&Peel	-0.09536	0.00034	PC2P < Core&Peel	0.05559	0.06015	-	-0.04530	0.10464	-
east		PC2P-IMHRC	-0.03025	0.63801	-	0.00523	1.00000	-	-0.00288	1.00000	-	-0.01491	0.99989	-	0.04045	0.67009	-	0.01887	0.99129	-

Supplementary Table 4. Resulst of the post-hoc Tukey test comparing the means of GO semantic similarity over the clusters for biological process (BP), molecular function (MF), and cellular compartment (CC). Results are show for min and mean analyses for four data sets of yeast and two data sets of human.

Tukey test			Mean								
			BP			MF			CC		
			diff	p.adj	Interpretation	diff	p.adj	Interpretation	diff	p.adj	Interpretation
Ye	KroganCore	MCODE-MCL	0.06343	0.13219	-	-0.00362	1.00000	-	0.02275	0.81707	-
		CFinder-MCL	0.00873	0.99999	-	-0.03550	0.61714	-	0.00001	1.00000	-
		AP-MCL	-0.10953	0.00000	AP < MCL	-0.10308	0.00000	AP < MCL	-0.06513	0.00000	AP < MCL
		CMC-MCL	0.01370	0.99982	-	-0.05272	0.22292	-	-0.00910	0.99963	-
		ClusterOne-MCL	-0.02499	0.85199	-	-0.05766	0.00270	ClusterOne < MCL	-0.02568	0.17628	-
		ProRank+-MCL	0.03253	0.41805	-	-0.05065	0.00538	ProRank+ < MCL	-0.00478	0.99994	-
		Core&Peel-MCL	0.12370	0.00000	Core&Peel > MCL	0.00144	1.00000	-	0.05261	0.00001	Core&Peel > MCL
		IMHRC-MCL	-0.14858	0.00000	IMHRC < MCL	-0.14372	0.00000	IMHRC < MCL	-0.07455	0.00000	IMHRC < MCL
		PC2P-MCL	-0.00821	0.99981	-	-0.02004	0.81912	-	-0.00074	1.00000	-
		CFinder-MCODE	-0.05470	0.48393	-	-0.03187	0.93675	-	-0.02274	0.90056	-
		AP-MCODE	-0.17296	0.00000	AP < MCODE	-0.09945	0.00005	AP < MCODE	-0.08788	0.00000	AP < MCODE
		CMC-MCODE	-0.04973	0.72381	-	-0.04910	0.63438	-	-0.03185	0.65770	-
		ClusterOne-MCODE	-0.08842	0.00341	ClusterOne < MCODE	-0.05404	0.21226	-	-0.04843	0.01446	ClusterOne < MCODE
		ProRank+-MCODE	-0.03090	0.91789	-	-0.04703	0.34777	-	-0.02753	0.52926	-
		Core&Peel-MCODE	0.06028	0.21878	-	0.00506	1.00000	-	0.02986	0.51436	-
		IMHRC-MCODE	-0.21201	0.00000	IMHRC < MCODE	-0.14010	0.00000	IMHRC < MCODE	-0.09730	0.00000	IMHRC < MCODE
		PC2P-MCODE	-0.07164	0.02026	PC2P < MCODE	-0.01642	0.99756	-	-0.02349	0.69523	-
		AP-CFinder	-0.11826	0.00000	AP < CFinder	-0.06758	0.00441	AP < CFinder	-0.06514	0.00000	AP < CFinder
		CMC-CFinder	0.00497	1.00000	-	-0.01723	0.99915	-	-0.00911	0.99986	-
		ClusterOne-CFinder	-0.03372	0.77623	-	-0.02216	0.96626	-	-0.02569	0.47480	-
		ProRank+-CFinder	0.02380	0.95591	-	-0.01515	0.99671	-	-0.00479	0.99999	-
		Core&Peel-CFinder	0.11497	0.00000	Core&Peel > CFinder	0.03693	0.61464	-	0.05259	0.00085	Core&Peel > CFinder
		IMHRC-CFinder	-0.15731	0.00000	IMHRC < CFinder	-0.10822	0.00000	IMHRC < CFinder	-0.07456	0.00000	IMHRC < CFinder
		PC2P-CFinder	-0.01695	0.99381	-	0.01546	0.99421	-	-0.00075	1.00000	-
		CMC-AP	0.12323	0.00000	CMC > AP	0.05035	0.25335	-	0.05603	0.00087	CMC > AP
		ClusterOne-AP	0.08454	0.00000	ClusterOne > AP	0.04542	0.03408	ClusterOne > AP	0.03945	0.00065	ClusterOne > AP
		ProRank+-AP	0.14206	0.00000	ProRank+ > AP	0.05243	0.00129	ProRank+ > AP	0.06035	0.00000	ProRank+ > AP
		Core&Peel-AP	0.23324	0.00000	Core&Peel > AP	0.10451	0.00000	Core&Peel > AP	0.11773	0.00000	Core&Peel > AP
		IMHRC-AP	-0.03905	0.09334	-	-0.04064	0.02975	IMHRC < AP	-0.00942	0.97669	-
		PC2P-AP	0.10132	0.00000	PC2P > AP	0.08304	0.00000	PC2P > AP	0.06439	0.00000	PC2P > AP
		ClusterOne-CMC	-0.03869	0.76360	-	-0.00494	1.00000	-	-0.01658	0.96620	-
		ProRank+-CMC	0.01883	0.99679	-	0.00208	1.00000	-	0.00432	1.00000	-
		Core&Peel-CMC	0.11000	0.00006	Core&Peel > CMC	0.05416	0.22654	-	0.06170	0.00037	Core&Peel > CMC
		IMHRC-CMC	-0.16228	0.00000	IMHRC < CMC	-0.09100	0.00010	IMHRC < CMC	-0.06545	0.00001	IMHRC < CMC
		PC2P-CMC	-0.02192	0.98671	-	0.03269	0.76745	-	0.00836	0.99963	-
		ProRank+-ClusterOne	0.05752	0.00242	ProRank+ > ClusterOne	0.00701	0.99995	-	0.02090	0.32323	-
		Core&Peel-ClusterOne	0.14869	0.00000	Core&Peel > ClusterOne	0.05910	0.00450	Core&Peel > ClusterOne	0.07829	0.00000	Core&Peel > ClusterOne
		IMHRC-ClusterOne	-0.12359	0.00000	IMHRC < ClusterOne	-0.08606	0.00000	IMHRC < ClusterOne	-0.04887	0.00000	IMHRC < ClusterOne
		PC2P-ClusterOne	0.01677	0.95631	-	0.03762	0.05407	-	0.02494	0.05178	-
		Core&Peel-ProRank+	0.09117	0.00000	Core&Peel > ProRank+	0.05209	0.00936	Core&Peel > ProRank+	0.05739	0.00000	Core&Peel > ProRank+
		IMHRC-ProRank+	-0.18111	0.00000	IMHRC < ProRank+	-0.09307	0.00000	IMHRC < ProRank+	-0.06977	0.00000	IMHRC < ProRank+
		PC2P-ProRank+	-0.04075	0.01387	PC2P < ProRank+	0.03061	0.10466	-	0.00404	0.99990	-
		IMHRC-Core&Peel	-0.27228	0.00000	IMHRC < Core&Peel	-0.14516	0.00000	IMHRC < Core&Peel	-0.12715	0.00000	IMHRC < Core&Peel
		PC2P-Core&Peel	-0.13192	0.00000	PC2P < Core&Peel	-0.02148	0.82866	-	-0.05334	0.00000	PC2P < Core&Peel
		PC2P-IMHRC	0.14036	0.00000	PC2P > IMHRC	0.12368	0.00000	PC2P > IMHRC	0.07381	0.00000	PC2P > IMHRC
	KroganExt	MCODE-MCL	0.05502	0.43699	-	-0.00333	1.00000	-	0.02365	0.90298	-
		CFinder-MCL	0.04859	0.07781	-	0.01148	0.99884	-	0.04913	0.00013	CFinder > MCL
		AP-MCL	-0.08929	0.00000	AP < MCL	-0.08289	0.00000	AP < MCL	-0.04182	0.00233	AP < MCL
		CMC-MCL	0.04706	0.09290	-	-0.02108	0.91161	-	0.02137	0.55736	-
		ClusterOne-MCL	0.04810	0.00347	ClusterOne > MCL	0.00612	0.99994	-	0.02194	0.15267	-
		ProRank+-MCL	0.12501	0.00000	ProRank+ > MCL	0.03540	0.11488	-	0.06006	0.00000	ProRank+ > MCL
		Core&Peel-MCL	0.14516	0.00000	Core&Peel > MCL	0.04604	0.00030	Core&Peel > MCL	0.07508	0.00000	Core&Peel > MCL
		IMHRC-MCL	-0.06983	0.00000	IMHRC < MCL	-0.07497	0.00000	IMHRC < MCL	-0.02838	0.00009	IMHRC < MCL
		PC2P-MCL	0.06056	0.00000	PC2P > MCL	0.04358	0.00000	PC2P > MCL	0.04196	0.00000	PC2P > MCL
		CFinder-MCODE	-0.00644	1.00000	-	0.01481	0.99990	-	0.02548	0.93043	-
		AP-MCODE	-0.14431	0.00001	AP < MCODE	-0.07956	0.05930	-	-0.06547	0.01264	AP < MCODE
		CMC-MCODE	-0.00797	1.00000	-	-0.01775	0.99956	-	-0.00229	1.00000	-
		ClusterOne-MCODE	-0.00692	1.00000	-	0.00945	1.00000	-	-0.00171	1.00000	-
		ProRank+-MCODE	0.06999	0.21112	-	0.03873	0.85489	-	0.03641	0.53439	-
		Core&Peel-MCODE	0.09013	0.01641	Core&Peel > MCODE	0.04937	0.52116	-	0.05142	0.06468	-
		IMHRC-MCODE	-0.12486	0.00002	IMHRC < MCODE	-0.07165	0.05057	-	-0.05203	0.04261	IMHRC < MCODE
		PC2P-MCODE	0.00554	1.00000	-	0.04691	0.53733	-	0.01830	0.98043	-
		AP-CFinder	-0.13788	0.00000	AP < CFinder	-0.09437	0.00004	AP < CFinder	-0.09095	0.00000	AP < CFinder
		CMC-CFinder	-0.00153	1.00000	-	-0.03255	0.79298	-	-0.02776	0.57200	-
		ClusterOne-CFinder	-0.00048	1.00000	-	-0.00536	1.00000	-	-0.02719	0.39757	-
		ProRank+-CFinder	0.07642	0.00274	ProRank+ > CFinder	0.02392	0.93653	-	0.01093	0.99714	-
		Core&Peel-CFinder	0.09657	0.00000	Core&Peel > CFinder	0.03456	0.48913	-	0.02594	0.41175	-
		IMHRC-CFinder	-0.11842	0.00000	IMHRC < CFinder	-0.08645	0.00000	IMHRC < CFinder	-0.07751	0.00000	IMHRC < CFinder
		PC2P-CFinder	0.01197	0.99921	-	0.03210	0.46423	-	-0.00717	0.99959	-
		CMC-AP	0.13634	0.00000	CMC > AP	0.06181	0.03777	CMC > AP	0.06318	0.00014	CMC > AP
		ClusterOne-AP	0.13739	0.00000	ClusterOne > AP	0.08901	0.00000	ClusterOne > AP	0.06376	0.00000	ClusterOne > AP
		ProRank+-AP	0.21430	0.00000	ProRank+ > AP	0.11829	0.00000	ProRank+ > AP	0.10187	0.00000	ProRank+ > AP
		Core&Peel-AP	0.23445	0.00000	Core&Peel > AP	0.12893	0.00000	Core&Peel > AP	0.11689	0.00000	Core&Peel > AP
		IMHRC-AP	0.01946	0.97243	-	0.00792	0.99995	-	0.01344	0.95935	-
		PC2P-AP	0.14985	0.00000	PC2P > AP	0.12647	0.00000	PC2P > AP	0.08377	0.00000	PC2P > AP
		ClusterOne-CMC	0.00105	1.00000	-	0.02720	0.82544	-	0.00058	1.00000	-
		ProRank+-CMC	0.07796	0.00174	ProRank+ > CMC	0.05647	0.03787	ProRank+ > CMC	0.03869	0.05617	-
		Core&Peel-CMC	0.09810	0.00000	Core&Peel > CMC	0.06712	0.00105	Core&Peel > CMC	0.05371	0.00010	Core&Peel > CMC
		IMHRC-CMC	-0.11689	0.00000	IMHRC < CMC	-0.05390	0.01009	IMHRC < CMC	-0.04975	0.00011	IMHRC < CMC
		PC2P-CMC	0.01351	0.99777	-	0.06466	0.00037	PC2P > CMC	0.02059	0.60700	-
		ProRank+-ClusterOne	0.07691	0.00007	ProRank+ > ClusterOne	0.02928	0.59711	-	0.03812	0.00986	ProRank+ > ClusterOne
		Core&Peel-ClusterOne	0.09705	0.00000	Core&Peel > ClusterOne	0.03992	0.06103	-	0.05313	0.00000	Core&Peel > ClusterOne
		IMHRC-ClusterOne	-0.11794	0.00000	IMHRC < ClusterOne	-0.08109	0.00000	IMHRC < ClusterOne	-0.05032	0.00000	IMHRC < ClusterOne
		PC2P-ClusterOne	0.01246	0.99100	-	0.03746	0.02624	PC2P > ClusterOne	0.02001	0.25577	-
		Core&Peel-ProRank+	0.02015	0.94930	-	0.01064	0.99902	-	0.01502	0.88758	-
		IMHRC-ProRank+	-0.19484	0.00000	IMHRC < ProRank+	-0.11037	0.00000	IMHRC < ProRank+	-0.08844	0.00000	IMHRC < ProRank+
		PC2P-ProRank+	-0.06445	0.00008	PC2P < ProRank+	0.00818	0.99968	-	-0.01810	0.55365	-
		IMHRC-Core&Peel	-0.21499	0.00000	IMHRC < Core&Peel	-0.12102	0.00000	IMHRC < Core&Peel	-0.10346	0.00000	IMHRC < Core&Peel
		PC2P-Core&Peel	-0.08460	0.00000	PC2P < Core&Peel	-0.00246	1.00000	-	-0.03312	0.00022	PC2P < Core&Peel
		PC2P-IMHRC	0.13039	0.00000	PC2P > IMHRC	0.11855	0.00000	PC2P > IMHRC	0.07034	0.00000	PC2P > IMHRC

Tukey test			Min								
			BP			MF			CC		
			diff	p.adj	Interpretation	diff	p.adj	Interpretation	diff	p.adj	Interpretation
Ye	KroganCore	MCODE-MCL	0.10962	0.09490	-	-0.04481	0.95413	-	0.04077	0.91824	-
		CFinder-MCL	-0.00772	1.00000	-	-0.09154	0.06783	-	-0.02073	0.99796	-
		AP-MCL	-0.13688	0.00000	AP < MCL	-0.15951	0.00000	AP < MCL	-0.13235	0.00000	AP < MCL
		CMC-MCL	-0.05165	0.92363	-	-0.16265	0.00007	CMC < MCL	-0.06930	0.28189	-
		ClusterOne-MCL	-0.09266	0.01323	ClusterOne < MCL	-0.15389	0.00000	ClusterOne < MCL	-0.09143	0.00019	ClusterOne < MCL
		ProRank+-MCL	0.01395	0.99989	-	-0.14408	0.00000	ProRank+ < MCL	-0.05781	0.04963	ProRank+ < MCL
		Core&Peel-MCL	0.16327	0.00000	Core&Peel > MCL	-0.07322	0.11666	-	0.08986	0.00092	Core&Peel > MCL
		IMHRC-MCL	-0.23129	0.00000	IMHRC < MCL	-0.24482	0.00000	IMHRC < MCL	-0.19638	0.00000	IMHRC < MCL
		PC2P-MCL	-0.01868	0.99752	-	-0.04156	0.55078	-	0.00098	1.00000	-
		CFinder-MCODE	-0.11734	0.13516	-	-0.04673	0.97147	-	-0.06150	0.65502	-
		AP-MCODE	-0.24650	0.00000	AP < MCODE	-0.11470	0.02416	AP < MCODE	-0.17312	0.00000	AP < MCODE
		CMC-MCODE	-0.16126	0.01353	CMC < MCODE	-0.11784	0.13182	-	-0.11007	0.04799	CMC < MCODE
		ClusterOne-MCODE	-0.20228	0.00000	ClusterOne < MCODE	-0.10908	0.04927	ClusterOne < MCODE	-0.13219	0.00015	ClusterOne < MCODE
		ProRank+-MCODE	-0.09567	0.18278	-	-0.09927	0.08036	-	-0.09857	0.01177	ProRank+ < MCODE
		Core&Peel-MCODE	0.05366	0.92778	-	-0.02842	0.99859	-	0.04909	0.81127	-
		IMHRC-MCODE	-0.34090	0.00000	IMHRC < MCODE	-0.20001	0.00000	IMHRC < MCODE	-0.23714	0.00000	IMHRC < MCODE
		PC2P-MCODE	-0.12830	0.00730	PC2P < MCODE	0.00325	1.00000	-	-0.03979	0.88693	-
		AP-CFinder	-0.12916	0.00162	AP < CFinder	-0.06797	0.36309	-	-0.11162	0.00015	AP < CFinder
		CMC-CFinder	-0.04393	0.98782	-	-0.07111	0.69370	-	-0.04857	0.87610	-
		ClusterOne-CFinder	-0.08494	0.19883	-	-0.06235	0.53156	-	-0.07069	0.11396	-
		ProRank+-CFinder	0.02167	0.99946	-	-0.05254	0.69504	-	-0.03708	0.85380	-
		Core&Peel-CFinder	0.17099	0.00002	Core&Peel > CFinder	0.01832	0.99988	-	0.11059	0.00072	Core&Peel > CFinder
		IMHRC-CFinder	-0.22357	0.00000	IMHRC < CFinder	-0.15328	0.00000	IMHRC < CFinder	-0.17565	0.00000	IMHRC < CFinder
		PC2P-CFinder	-0.01096	1.00000	-	0.04998	0.68895	-	0.02171	0.99323	-
		CMC-AP	0.08523	0.33605	-	-0.00314	1.00000	-	0.06305	0.38701	-
		ClusterOne-AP	0.04421	0.74560	-	0.00562	1.00000	-	0.04092	0.48715	-
		ProRank+-AP	0.15082	0.00000	ProRank+ > AP	0.01543	0.99926	-	0.07454	0.00070	ProRank+ > AP
		Core&Peel-AP	0.30015	0.00000	Core&Peel > AP	0.08628	0.01643	Core&Peel > AP	0.22220	0.00000	Core&Peel > AP
		IMHRC-AP	-0.09441	0.00071	IMHRC < AP	-0.08531	0.00111	IMHRC < AP	-0.06403	0.00533	IMHRC < AP
		PC2P-AP	0.11820	0.00000	PC2P > AP	0.11795	0.00000	PC2P > AP	0.13333	0.00000	PC2P > AP
		ClusterOne-CMC	-0.04102	0.98220	-	0.00876	1.00000	-	-0.02212	0.99869	-
		ProRank+-CMC	0.06559	0.68614	-	0.01857	0.99991	-	0.01150	0.99999	-
		Core&Peel-CMC	0.21492	0.00000	Core&Peel > CMC	0.08942	0.23536	-	0.15916	0.00000	Core&Peel > CMC
		IMHRC-CMC	-0.17964	0.00001	IMHRC < CMC	-0.08217	0.23309	-	-0.12707	0.00007	IMHRC < CMC
		PC2P-CMC	0.03297	0.99327	-	0.12109	0.00397	PC2P > CMC	0.07028	0.16041	-
		ProRank+-ClusterOne	0.10661	0.00029	ProRank+ > ClusterOne	0.00981	0.99999	-	0.03362	0.69840	-
		Core&Peel-ClusterOne	0.25594	0.00000	Core&Peel > ClusterOne	0.08067	0.04922	Core&Peel > ClusterOne	0.18128	0.00000	Core&Peel > ClusterOne
		IMHRC-ClusterOne	-0.13862	0.00000	IMHRC < ClusterOne	-0.09093	0.00085	IMHRC < ClusterOne	-0.10495	0.00000	IMHRC < ClusterOne
		PC2P-ClusterOne	0.07398	0.02101	PC2P > ClusterOne	0.11233	0.00000	PC2P > ClusterOne	0.09240	0.00000	PC2P > ClusterOne
		Core&Peel-ProRank+	0.14933	0.00000	Core&Peel > ProRank+	0.07086	0.08040	-	0.14766	0.00000	Core&Peel > ProRank+
		IMHRC-ProRank+	-0.24523	0.00000	IMHRC < ProRank+	-0.10074	0.00001	IMHRC < ProRank+	-0.13857	0.00000	IMHRC < ProRank+
		PC2P-ProRank+	-0.03263	0.78196	-	0.10252	0.00000	PC2P > ProRank+	0.05878	0.00207	PC2P > ProRank+
		IMHRC-Core&Peel	-0.39456	0.00000	IMHRC < Core&Peel	-0.17159	0.00000	IMHRC < Core&Peel	-0.28623	0.00000	IMHRC < Core&Peel
		PC2P-Core&Peel	-0.18195	0.00000	PC2P < Core&Peel	0.03166	0.90978	-	-0.08888	0.00004	PC2P < Core&Peel
		PC2P-IMHRC	0.21261	0.00000	PC2P > IMHRC	0.20326	0.00000	PC2P > IMHRC	0.19735	0.00000	PC2P > IMHRC
	KroganExt	MCODE-MCL	0.09066	0.38549	-	-0.02477	0.99966	-	0.03507	0.98644	-
		CFinder-MCL	0.06528	0.24646	-	-0.01170	0.99998	-	0.08602	0.00201	CFinder > MCL
		AP-MCL	-0.06063	0.33476	-	-0.09504	0.00276	AP < MCL	-0.09577	0.00022	AP < MCL
		CMC-MCL	0.01657	0.99973	-	-0.07966	0.02891	CMC < MCL	0.00108	1.00000	-
		ClusterOne-MCL	0.00617	1.00000	-	-0.06069	0.03119	ClusterOne < MCL	-0.01376	0.99762	-
		ProRank+-MCL	0.15418	0.00000	ProRank+ > MCL	0.00000	1.00000	-	0.08508	0.00008	ProRank+ > MCL
		Core&Peel-MCL	0.18154	0.00000	Core&Peel > MCL	-0.01686	0.99194	-	0.12108	0.00000	Core&Peel > MCL
		IMHRC-MCL	-0.11659	0.00000	IMHRC < MCL	-0.13559	0.00000	IMHRC < MCL	-0.10237	0.00000	IMHRC < MCL
		PC2P-MCL	0.07196	0.00001	PC2P > MCL	0.05972	0.00017	PC2P > MCL	0.08473	0.00000	PC2P > MCL
		CFinder-MCODE	-0.02538	0.99992	-	0.01307	1.00000	-	0.05094	0.93412	-
		AP-MCODE	-0.15129	0.02519	AP < MCODE	-0.07027	0.80960	-	-0.13084	0.01426	AP < MCODE
		CMC-MCODE	-0.07409	0.81945	-	-0.05489	0.95193	-	-0.03400	0.99578	-
		ClusterOne-MCODE	-0.08449	0.57833	-	-0.03592	0.99588	-	-0.04883	0.92030	-
		ProRank+-MCODE	0.06352	0.89653	-	0.02477	0.99982	-	0.05001	0.92026	-
		Core&Peel-MCODE	0.09088	0.44155	-	0.00791	1.00000	-	0.08601	0.24144	-
		IMHRC-MCODE	-0.20724	0.00001	IMHRC < MCODE	-0.11082	0.08425	-	-0.13745	0.00107	IMHRC < MCODE
		PC2P-MCODE	-0.01869	0.99998	-	0.08449	0.39368	-	0.04966	0.87854	-
		AP-CFinder	-0.12591	0.00630	AP < CFinder	-0.08334	0.18922	-	-0.18179	0.00000	AP < CFinder
		CMC-CFinder	-0.04872	0.90745	-	-0.06796	0.47658	-	-0.08494	0.06263	-
		ClusterOne-CFinder	-0.05911	0.57695	-	-0.04899	0.73891	-	-0.09977	0.00135	ClusterOne < CFinder
		ProRank+-CFinder	0.08890	0.10234	-	0.01170	0.99999	-	-0.00094	1.00000	-
		Core&Peel-CFinder	0.11625	0.00142	Core&Peel > CFinder	-0.00517	1.00000	-	0.03507	0.88538	-
		IMHRC-CFinder	-0.18187	0.00000	IMHRC < CFinder	-0.12390	0.00002	IMHRC < CFinder	-0.18839	0.00000	IMHRC < CFinder
		PC2P-CFinder	0.00668	1.00000	-	0.07142	0.08721	-	-0.00128	1.00000	-
		CMC-AP	0.07719	0.37060	-	0.01538	0.99997	-	0.09685	0.01456	CMC > AP
		ClusterOne-AP	0.06680	0.38010	-	0.03434	0.96057	-	0.08201	0.02038	ClusterOne > AP
		ProRank+-AP	0.21481	0.00000	ProRank+ > AP	0.09504	0.02865	ProRank+ > AP	0.18085	0.00000	ProRank+ > AP
		Core&Peel-AP	0.24216	0.00000	Core&Peel > AP	0.07817	0.08105	-	0.21685	0.00000	Core&Peel > AP
		IMHRC-AP	-0.05596	0.47951	-	-0.04056	0.80976	-	-0.00661	1.00000	-
		PC2P-AP	0.13259	0.00001	PC2P > AP	0.15475	0.00000	PC2P > AP	0.18050	0.00000	PC2P > AP
		ClusterOne-CMC	-0.01040	1.00000	-	0.01896	0.99952	-	-0.01483	0.99981	-
		ProRank+-CMC	0.13762	0.00025	ProRank+ > CMC	0.07966	0.13572	-	0.08400	0.02681	ProRank+ > CMC
		Core&Peel-CMC	0.16497	0.00000	Core&Peel > CMC	0.06279	0.32064	-	0.12000	0.00001	Core&Peel > CMC
		IMHRC-CMC	-0.13315	0.00001	IMHRC < CMC	-0.05594	0.38256	-	-0.10345	0.00005	IMHRC < CMC
		PC2P-CMC	0.05540	0.46699	-	0.13937	0.00000	PC2P > CMC	0.08366	0.00256	PC2P > CMC
		ProRank+-ClusterOne	0.14801	0.00000	ProRank+ > ClusterOne	0.06069	0.25093	-	0.09884	0.00012	ProRank+ > ClusterOne
		Core&Peel-ClusterOne	0.17536	0.00000	Core&Peel > ClusterOne	0.04383	0.54489	-	0.13484	0.00000	Core&Peel > ClusterOne
		IMHRC-ClusterOne	-0.12276	0.00000	IMHRC < ClusterOne	-0.07490	0.00273	IMHRC < ClusterOne	-0.08862	0.00000	IMHRC < ClusterOne
		PC2P-ClusterOne	0.06579	0.02473	PC2P > ClusterOne	0.12041	0.00000	PC2P > ClusterOne	0.09849	0.00000	PC2P > ClusterOne
		Core&Peel-ProRank+	0.02735	0.98222	-	-0.01686	0.99925	-	0.03600	0.73745	-
		IMHRC-ProRank+	-0.27077	0.00000	IMHRC < ProRank+	-0.13559	0.00000	IMHRC < ProRank+	-0.18746	0.00000	IMHRC < ProRank+
		PC2P-ProRank+	-0.08222	0.00514	PC2P < ProRank+	0.05972	0.08918	-	-0.00035	1.00000	-
		IMHRC-Core&Peel	-0.29812	0.00000	IMHRC < Core&Peel	-0.11873	0.00000	IMHRC < Core&Peel	-0.22346	0.00000	IMHRC < Core&Peel
		PC2P-Core&Peel	-0.10957	0.00000	PC2P < Core&Peel	0.07658	0.00019	PC2P > Core&Peel	-0.03635	0.27820	-
		PC2P-IMHRC	0.18855	0.00000	PC2P > IMHRC	0.19531	0.00000	PC2P > IMHRC	0.18711	0.00000	PC2P > IMHRC

Supplementary Table 4. Resulst of the post-hoc Tukey test comparing the means of GO semantic similarity over the clusters for biological process (BP), molecular function (MF), and cellular compartment (CC). Results are show for min and mean analyses for four data sets of yeast and two data sets of human.

Tukey test			Mean									Min								
			BP			MF			CC			BP			MF			CC		
			diff	p.adj	Interpretation	diff	p.adj	Interpretation	diff	p.adj	Interpretation	diff	p.adj	Interpretation	diff	p.adj	Interpretation	diff	p.adj	Interpretation
Human	PIPS	MCODE-MCL	-0.06849	0.08158	-	-0.05738	0.09142	-	-0.04211	0.33398	-	-0.07605	0.41565	-	-0.09198	0.26049	-	-0.07008	0.50211	-
		CFinder-MCL	-0.06717	0.06107	-	-0.04970	0.17439	-	-0.03429	0.56378	-	-0.10203	0.04568	CFinder < MCL	-0.09783	0.12994	-	-0.07360	0.34756	-
		AP-MCL	-0.07751	0.00067	AP < MCL	-0.05887	0.00447	AP < MCL	-0.05101	0.00877	AP < MCL	-0.10747	0.00196	AP < MCL	-0.10749	0.00699	AP < MCL	-0.09861	0.00509	AP < MCL
		CMC-MCL	-0.09924	0.00001	CMC < MCL	-0.07911	0.00005	CMC < MCL	-0.08797	0.00000	CMC < MCL	-0.08999	0.04615	CMC < MCL	-0.13156	0.00076	CMC < MCL	-0.14014	0.00002	CMC < MCL
		ClusterOne-MCL	-0.14146	0.00000	ClusterOne < MCL	-0.10867	0.00000	ClusterOne < MCL	-0.08490	0.00000	ClusterOne < MCL	-0.18280	0.00000	ClusterOne < MCL	-0.17682	0.00000	ClusterOne < MCL	-0.15125	0.00000	ClusterOne < MCL
		ProRank+-MCL	-0.08081	0.00003	ProRank+ < MCL	-0.08481	0.00000	ProRank+ < MCL	-0.06192	0.00003	ProRank+ < MCL	-0.09495	0.00270	ProRank+ < MCL	-0.15070	0.00000	ProRank+ < MCL	-0.10484	0.00028	ProRank+ < MCL
		Core&Peel-MCL	-0.11743	0.00000	Core&Peel < MCL	-0.09369	0.00000	Core&Peel < MCL	-0.11691	0.00000	Core&Peel < MCL	-0.11932	0.00009	Core&Peel < MCL	-0.15810	0.00000	Core&Peel < MCL	-0.18794	0.00000	Core&Peel < MCL
		IMHRC-MCL	-0.14097	0.00000	IMHRC < MCL	-0.11554	0.00000	IMHRC < MCL	-0.07907	0.00000	IMHRC < MCL	-0.18178	0.00000	IMHRC < MCL	-0.20442	0.00000	IMHRC < MCL	-0.14896	0.00000	IMHRC < MCL
		PC2P-MCL	-0.03417	0.57774	-	-0.03024	0.51767	-	-0.02752	0.51514	-	-0.05789	0.36651	-	-0.04873	0.72962	-	-0.05127	0.51283	-
		CFinder-MCODE	0.00132	1.00000	-	0.00768	1.00000	-	0.00782	0.99999	-	-0.02597	0.99914	-	-0.00585	1.00000	-	-0.00352	1.00000	-
		AP-MCODE	-0.00901	0.99999	-	-0.00148	1.00000	-	-0.00891	0.99991	-	-0.03142	0.98798	-	-0.01551	0.99998	-	-0.02854	0.99279	-
		CMC-MCODE	-0.03075	0.90975	-	-0.02173	0.97105	-	-0.04586	0.13404	-	-0.01394	0.99999	-	-0.03959	0.97680	-	-0.07006	0.38475	-
		ClusterOne-MCODE	-0.07296	0.00418	ClusterOne < MCODE	-0.05128	0.04347	ClusterOne < MCODE	-0.04279	0.09261	-	-0.10675	0.00460	ClusterOne < MCODE	-0.08485	0.12937	-	-0.08117	0.07894	-
		ProRank+-MCODE	-0.01232	0.99968	-	-0.02743	0.76864	-	-0.01981	0.93106	-	-0.01890	0.99955	-	-0.05872	0.60983	-	-0.03476	0.95171	-
		Core&Peel-MCODE	-0.04893	0.24843	-	-0.03631	0.44518	-	-0.07481	0.00002	Core&Peel < MCODE	-0.04327	0.88251	-	-0.06612	0.49648	-	-0.11787	0.00090	Core&Peel < MCODE
		IMHRC-MCODE	-0.07248	0.00832	IMHRC < MCODE	-0.05815	0.01731	IMHRC < MCODE	-0.03696	0.29895	-	-0.10572	0.00944	IMHRC < MCODE	-0.11244	0.01250	IMHRC < MCODE	-0.07888	0.13475	-
		PC2P-MCODE	0.03432	0.73716	-	0.02715	0.81059	-	0.01459	0.99287	-	0.01817	0.99975	-	0.04324	0.92270	-	0.01881	0.99959	-
		AP-CFinder	-0.01033	0.99993	-	-0.00916	0.99990	-	-0.01673	0.97824	-	-0.00544	1.00000	-	-0.00966	1.00000	-	-0.02502	0.99545	-
		CMC-CFinder	-0.03207	0.84163	-	-0.02941	0.77131	-	-0.05368	0.01716	CMC < CFinder	0.01203	0.99999	-	-0.03374	0.98821	-	-0.06654	0.36854	-
		ClusterOne-CFinder	-0.07428	0.00080	ClusterOne < CFinder	-0.05896	0.00259	ClusterOne < CFinder	-0.05061	0.00616	ClusterOne < CFinder	-0.08077	0.05033	-	-0.07900	0.12125	-	-0.07765	0.05751	-
		ProRank+-CFinder	-0.01364	0.99857	-	-0.03511	0.30864	-	-0.02763	0.52463	-	0.00707	1.00000	-	-0.05287	0.63978	-	-0.03124	0.95835	-
		Core&Peel-CFinder	-0.05026	0.13249	-	-0.04399	0.10654	-	-0.08263	0.00000	Core&Peel < CFinder	-0.01730	0.99971	-	-0.06027	0.52150	-	-0.11434	0.00037	Core&Peel < CFinder
		IMHRC-CFinder	-0.07380	0.00208	IMHRC < CFinder	-0.06583	0.00089	IMHRC < CFinder	-0.04478	0.04667	IMHRC < CFinder	-0.07975	0.08600	-	-0.10659	0.00961	IMHRC < CFinder	-0.07536	0.11004	-
		PC2P-CFinder	0.03300	0.69114	-	0.01947	0.95454	-	0.00677	0.99997	-	0.04414	0.79771	-	0.04910	0.77447	-	0.02233	0.99704	-
		CMC-AP	-0.02174	0.93191	-	-0.02025	0.88501	-	-0.03695	0.07034	-	0.01748	0.99910	-	-0.02407	0.99436	-	-0.04153	0.70758	-
		ClusterOne-AP	-0.06395	0.00001	ClusterOne < AP	-0.04980	0.00009	ClusterOne < AP	-0.03389	0.01372	ClusterOne < AP	-0.07533	0.00141	ClusterOne < AP	-0.06933	0.01611	ClusterOne < AP	-0.05263	0.08664	-
		ProRank+-AP	-0.00331	1.00000	-	-0.02595	0.23034	-	-0.01090	0.97384	-	0.01252	0.99942	-	-0.04321	0.40502	-	-0.00622	1.00000	-
		Core&Peel-AP	-0.03992	0.06887	-	-0.03483	0.05427	-	-0.06590	0.00000	Core&Peel < AP	-0.01186	0.99983	-	-0.05061	0.31274	-	-0.08933	0.00009	Core&Peel < AP
		IMHRC-AP	-0.06346	0.00012	IMHRC < AP	-0.05667	0.00004	IMHRC < AP	-0.02806	0.17676	-	-0.07431	0.00724	IMHRC < AP	-0.09693	0.00032	IMHRC < AP	-0.05035	0.21873	-
		PC2P-AP	0.04333	0.02492	PC2P > AP	0.02863	0.20196	-	0.02349	0.33845	-	0.04958	0.20287	-	0.05876	0.11430	-	0.04735	0.22846	-
		ClusterOne-CMC	-0.04221	0.07918	-	-0.02955	0.28031	-	0.00307	1.00000	-	-0.09281	0.00032	ClusterOne < CMC	-0.04526	0.58785	-	-0.01110	0.99993	-
		ProRank+-CMC	0.01843	0.94123	-	-0.00570	0.99997	-	0.02605	0.28261	-	-0.00496	1.00000	-	-0.01914	0.99712	-	0.03530	0.73325	-
		Core&Peel-CMC	-0.01819	0.96612	-	-0.01458	0.97691	-	-0.02895	0.24075	-	-0.02933	0.93992	-	-0.02654	0.98173	-	-0.04780	0.41114	-
		IMHRC-CMC	-0.04173	0.14806	-	-0.03643	0.12255	-	0.00890	0.99902	-	-0.09179	0.00151	IMHRC < CMC	-0.07286	0.07651	-	-0.00882	1.00000	-
		PC2P-CMC	0.06507	0.00030	PC2P > CMC	0.04887	0.00284	PC2P > CMC	0.06045	0.00000	PC2P > CMC	0.03210	0.88882	-	0.08283	0.01280	PC2P > CMC	0.08887	0.00080	PC2P > CMC
		ProRank+-ClusterOne	0.06064	0.00000	ProRank+ > ClusterOne	0.02385	0.09614	-	0.02298	0.05841	-	0.08785	0.00000	ProRank+ > ClusterOne	0.02612	0.79102	-	0.04641	0.02548	ProRank+ > ClusterOne
		Core&Peel-ClusterOne	0.02403	0.46840	-	0.01497	0.84759	-	-0.03201	0.00649	Core&Peel < ClusterOne	0.06347	0.00367	Core&Peel > ClusterOne	0.01872	0.98800	-	-0.03670	0.37458	-
		IMHRC-ClusterOne	0.00048	1.00000	-	-0.00687	0.99951	-	0.00583	0.99972	-	0.00102	1.00000	-	-0.02760	0.89353	-	0.00228	1.00000	-
		PC2P-ClusterOne	0.10728	0.00000	PC2P > ClusterOne	0.07843	0.00000	PC2P > ClusterOne	0.05738	0.00000	PC2P > ClusterOne	0.12491	0.00000	PC2P > ClusterOne	0.12809	0.00000	PC2P > ClusterOne	0.09998	0.00000	PC2P > ClusterOne
		Core&Peel-ProRank+	-0.03661	0.01772	Core&Peel < ProRank+	-0.00888	0.99252	-	-0.05500	0.00000	Core&Peel < ProRank+	-0.02437	0.85904	-	-0.00740	0.99999	-	-0.08311	0.00000	Core&Peel < ProRank+
		IMHRC-ProRank+	-0.06016	0.00000	IMHRC < ProRank+	-0.03072	0.03553	IMHRC < ProRank+	-0.01715	0.58894	-	-0.08683	0.00000	IMHRC < ProRank+	-0.05372	0.07170	-	-0.04412	0.14182	-
		PC2P-ProRank+	0.04664	0.00020	PC2P > ProRank+	0.05458	0.00000	PC2P > ProRank+	0.03440	0.00051	PC2P > ProRank+	0.03706	0.28084	-	0.10197	0.00000	PC2P > ProRank+	0.05357	0.00927	PC2P > ProRank+
		IMHRC-Core&Peel	-0.02354	0.65992	-	-0.02184	0.53503	-	0.03784	0.00281	IMHRC > Core&Peel	-0.06245	0.02049	IMHRC < Core&Peel	-0.04632	0.35535	-	0.03898	0.45257	-
		PC2P-Core&Peel	0.08325	0.00000	PC2P > Core&Peel	0.06346	0.00000	PC2P > Core&Peel	0.08939	0.00000	PC2P > Core&Peel	0.06144	0.01126	PC2P > Core&Peel	0.10937	0.00000	PC2P > Core&Peel	0.13668	0.00000	PC2P > Core&Peel
		PC2P-IMHRC	0.10680	0.00000	PC2P > IMHRC	0.08530	0.00000	PC2P > IMHRC	0.05155	0.00000	PC2P > IMHRC	0.12389	0.00000	PC2P > IMHRC	0.15569	0.00000	PC2P > IMHRC	0.09769	0.00000	PC2P > IMHRC
	STRING	MCODE-MCL	-0.01456	0.96288	-	0.00111	1.00000	-	-0.00066	1.00000	-	-0.04984	0.28792	-	-0.05509	0.24432	-	-0.01357	0.99769	-
		CFinder-MCL	-0.04934	0.00063	CFinder < MCL	-0.01077	0.98602	-	-0.01846	0.24352	-	-0.10112	0.00002	CFinder < MCL	-0.08609	0.00211	CFinder < MCL	-0.05438	0.01818	CFinder < MCL
		AP-MCL	0.00247	1.00000	-	-0.00195	1.00000	-	-0.00087	1.00000	-	0.00740	0.99995	-	0.00310	1.00000	-	0.00131	1.00000	-
		CMC-MCL	0.00193	1.00000	-	-0.00065	1.00000	-	-0.00030	1.00000	-	-0.04117	0.44244	-	-0.10269	0.00001	CMC < MCL	-0.02134	0.90673	-
		ClusterOne-MCL	-0.06464	0.00000	ClusterOne < MCL	-0.03125	0.00076	ClusterOne < MCL	-0.03060	0.00000	ClusterOne < MCL	-0.10350	0.00000	ClusterOne < MCL	-0.09202	0.00000	ClusterOne < MCL	-0.06627	0.00000	ClusterOne < MCL
		ProRank+-MCL	-0.02157	0.04419	ProRank+ < MCL	-0.02340	0.00264	ProRank+ < MCL	-0.01700	0.00295	ProRank+ < MCL	-0.09767	0.00000	ProRank+ < MCL	-0.14961	0.00000	ProRank+ < MCL	-0.06345	0.00000	ProRank+ < MCL
		Core&Peel-MCL	0.00773	0.99706	-	0.00048	1.00000	-	0.00525	0.99523	-	-0.05262	0.02150	Core&Peel < MCL	-0.12751	0.00000	Core&Peel < MCL	-0.02552	0.51755	-
		IMHRC-MCL	-0.05797	0.00000	IMHRC < MCL	-0.04052	0.00001	IMHRC < MCL	-0.02835	0.00003	IMHRC < MCL	-0.09178	0.00000	IMHRC < MCL	-0.10838	0.00000	IMHRC < MCL	-0.05737	0.00012	IMHRC < MCL
		PC2P-MCL	-0.00072	1.00000	-	0.00049	1.00000	-	0.00002	1.00000	-	-0.00199	1.00000	-	-0.00325	1.00000	-	-0.00019	1.00000	-
		CFinder-MCODE	-0.03478	0.32867	-	-0.01187	0.99516	-	-0.01781	0.65101	-	-0.05128	0.58153	-	-0.03100	0.98009	-	-0.04081	0.56516	-
		AP-MCODE	0.01703	0.92001	-	-0.00306	1.00000	-	-0.00021	1.00000	-	0.05724	0.15704	-	0.05819	0.21506	-	0.01488	0.99631	-
		CMC-MCODE	0.01649	0.97494	-	-0.00176	1.00000	-	0.00036	1.00000	-	0.00867	1.00000	-	-0.04760	0.71604	-	-0.00777	1.00000	-
		ClusterOne-MCODE	-0.05008	0.00194	ClusterOne < MCODE	-0.03236	0.07576	-	-0.02994	0.00523	ClusterOne < MCODE	-0.05365	0.26818	-	-0.03693	0.84430	-	-0.05270	0.05707	-
		ProRank+-MCODE	-0.00701	0.99982	-	-0.02451	0.26671	-	-0.01635	0.40089	-	-0.04783	0.30364	-	-0.09452	0.00030	ProRank+ < MCODE	-0.04989	0.04104	ProRank+ < MCODE
		Core&Peel-MCODE	0.02229	0.75377	-	-0.00063	1.00000	-	0.00590	0.99927	-	-0.00278	1.00000	-	-0.07242	0.06418	-	-0.01195	0.99957	-
		IMHRC-MCODE	-0.04341	0.02162	IMHRC < MCODE	-0.04163	0.00622	IMHRC < MCODE	-0.02770	0.02116	IMHRC < MCODE	-0.04194	0.66986	-	-0.05329	0.41925	-	-0.04380	0.25791	-
		PC2P-MCODE	0.01384	0.97422	-	-0.00061	1.00000	-	0.00067	1.00000	-	0.04786	0.35295	-	0.05184	0.33493	-	0.01338	0.99800	-
		AP-CFinder	0.05181	0.00046	AP > CFinder	0.00881	0.99751	-	0.01760	0.35681	-	0.10852	0.00001	AP > CFinder	0.08919	0.00202	AP > CFinder	0.05570	0.02017	AP > CFinder
		CMC-CFinder	0.05127	0.00756	CMC > CFinder	0.01012	0.99785	-	0.01817	0.54674	-	0.05996	0.27002	-	-0.01660	0.99975	-	0.03305	0.76697	-
		ClusterOne-CFinder	-0.01530	0.96128	-	-0.02048	0.63816	-	-0.01213	0.86070	-	-0.00237	1.00000	-	-0.00593	1.00000	-	-0.01189	0.99943	-
		ProRank+-CFinder	0.02777	0.25939	-	-0.01263	0.95027	-	0.00146	1.00000	-	0.00345	1.00000	-	-0.06352	0.06362	-	-0.00907	0.99987	-
		Core&Peel-CFinder	0.05707	0.00019	Core&Peel > CFinder	0.01124	0.98965	-	0.02371	0.08001	-	0.04851	0.42776	-	-0.04142	0.74217	-	0.02887	0.79823	-
		IMHRC-CFinder	-0.00863	0.99956	-	-0.02975	0.15759	-	-0.00989	0.96496	-	0.00934	0.99999	-	-0.02228	0.99443	-	-0.00299	1.00000	-
		PC2P-CFinder	0.04861	0.00091	PC2P > CFinder	0.01126	0.98154	-	0.01848	0.24842	-	0.09914	0.00003	PC2P > CFinder	0.08285	0.00418	PC2P > CFinder	0.05419	0.02002	PC2P > CFinder
		CMC-AP	-0.00054	1.00000	-	0.00130	1.00000	-	0.00057	1.00000	-	-0.04857	0.25578	-	-0.10579	0.00001	CMC < AP	-0.02265	0.89424	-
		ClusterOne-AP	-0.06711	0.00000	ClusterOne < AP	-0.02930	0.00586	ClusterOne < AP	-0.02973	0.00001	ClusterOne < AP	-0.11090	0.00000	ClusterOne < AP	-0.09512	0.00000	ClusterOne < AP	-0.06759	0.00000	ClusterOne < AP
		ProRank+-AP	-0.02404	0.03614	ProRank+ < AP	-0.02145	0.02778	ProRank+ < AP	-0.01613	0.02030	ProRank+ < AP	-0.10507	0.00000	ProRank+ < AP	-0.15271	0.00000	ProRank+ < AP	-0.06477	0.00000	ProRank+ < AP
		Core&Peel-AP	0.00526	0.99992	-	0.00243	1.00000	-	0.00612	0.99011	-	-0.06002	0.00805	Core&Peel < AP	-0.13061	0.00000	Core&Peel < AP	-0.02683	0.52270	-
		IMHRC-AP	-0.06044	0.00000	IMHRC < AP	-0.03857	0.00010	IMHRC < AP	-0.02748	0.00018	IMHRC < AP	-0.09918	0.00000	IMHRC < AP	-0.11148	0.00000	IMHRC < AP	-0.05868	0.00022	IMHRC < AP
		PC2P-AP	-0.00319	1.00000	-	0.00244	1.00000	-	0.00088	1.00000	-	-0.00939	0.99963	-	-0.00635	0.99999	-	-0.00150	1.00000	-
		ClusterOne-CMC	-0.06657	0.00000	ClusterOne < CMC	-0.03060	0.06331	-	-0.03030	0.00126	ClusterOne < CMC	-0.06233	0.05471	-	0.01067	0.99997	-	-0.04494	0.11367	-
		ProRank+-CMC	-0.02350	0.39413	-	-0.02275	0.24230	-	-0.01670	0.23788	-	-0.05651	0.05094	-	-0.04692	0.29543	-	-0.04212	0.08239	-
		Core&Peel-CMC	0.00581	0.99997	-	0.00113	1.00000	-	0.00555	0.99921	-	-0.01145	0.99992	-	-0.02482	0.98108	-	-0.00418	1.00000	-
		IMHRC-CMC	-0.05990	0.00002	IMHRC < CMC	-0.03987	0.00407	IMHRC < CMC	-0.02805	0.00720	IMHRC < CMC	-0.05061	0.28952	-	-0.00568	1.00000	-	-0.03603	0.43793	-
		PC2P-CMC	-0.00265	1.00000	-	0.00114	1.00000	-	0.00031	1.00000	-	0.03918	0.52582	-	0.09945	0.00003	PC2P > CMC	0.02115	0.91404	-
		ProRank+-ClusterOne	0.04307	0.00000	ProRank+ > ClusterOne	0.00785	0.98010	-	0.01360	0.17073	-	0.00582	0.99999	-	-0.05759	0.00369	ProRank+ < ClusterOne	0.00282	1.00000	-
		Core&Peel-ClusterOne	0.07237	0.00000	Core&Peel > ClusterOne	0.03173	0.00675	Core&Peel > ClusterOne	0.03585	0.00000	Core&Peel > ClusterOne	0.05088	0.07970	-	-0.03549	0.62823	-	0.04076	0.06847	-
		IMHRC-ClusterOne	0.00667	0.99962	-	-0.00927	0.98598	-	0.00225	1.00000	-	0.01171	0.99959	-	-0.01636	0.99666	-	0.00890	0.99970	-
		PC2P-ClusterOne	0.06391	0.00000	PC2P > ClusterOne	0.03174	0.00067	PC2P > ClusterOne	0.03061	0.00000	PC2P > ClusterOne	0.10151	0.00000	PC2P > ClusterOne	0.08877	0.00000	PC2P > ClusterOne	0.06608	0.00000	PC2P > ClusterOne
		Core&Peel-ProRank+	0.02930	0.01607	Core&Peel > ProRank+	0.02388	0.03386	Core&Peel > ProRank+	0.02225	0.00117	Core&Peel > ProRank+	0.04505	0.06100	-	0.02210	0.92113	-	0.03794	0.03138	Core&Peel > ProRank+
		IMHRC-ProRank+	-0.03640	0.00069	IMHRC < ProRank+	-0.01712	0.36885	-	-0.01135	0.51857	-	0.00589	1.00000	-	0.04124	0.20938	-	0.00609	0.99996	-
		PC2P-ProRank+	0.02084	0.06861	-	0.02389	0.00233	PC2P > ProRank+	0.01702	0.00358	PC2P > ProRank+	0.09569	0.00000	PC2P > ProRank+	0.14637	0.00000	PC2P > ProRank+	0.06326	0.00000	PC2P > ProRank+
		IMHRC-Core&Peel	-0.06570	0.00000	IMHRC < Core&Peel	-0.04100	0.00016	IMHRC < Core&Peel	-0.03360	0.00001	IMHRC < Core&Peel	-0.03916	0.45022	-	0.01914	0.99180	-	-0.03185	0.39994	-
		PC2P-Core&Peel	-0.00846	0.99454	-	0.00001	1.00000	-	-0.00523	0.99561	-	0.05063	0.03564	PC2P > Core&Peel	0.12427	0.00000	PC2P > Core&Peel	0.02533	0.54048	-
		PC2P-IMHRC	0.05724	0.00000	PC2P > IMHRC	0.04101	0.00001	PC2P > IMHRC	0.02837	0.00003	PC2P > IMHRC	0.08980	0.00000	PC2P > IMHRC	0.10513	0.00000	PC2P > IMHRC	0.05718	0.00015	PC2P > IMHRC

Supplementary Table 5. Empirical running time on the four PPI networks of yeast and the two PPI networks of human.

Yeast	Empirical time
Collins	0 hour, 8 minutes and 43 seconds
Gavin	0 hours, 32 minutes and 0 seconds
KroganCore	0 days, 4 hours, 31 minutes and 35 seconds
KroganExt	1 day, 13 hours, 20 minutes and 31 seconds

Human	Empirical time
PIPS	0 hours, 18 minutes and 2 seconds
STRING	0 hours, 5 minutes and 18 seconds

Supplementary Table 6. The average of enrichment score of predicted complexes with at least one enriched annotation over all clusters are compared for the nine approaches across all datasets.

Yeast

	method	BP	MF	CC
Collins	MCL	0.818	0.376	0.545
	MCODE	0.937	0.559	0.649
	CFinder	0.956	0.500	0.667
	AP	0.817	0.396	0.536
	CMC	0.989	0.645	0.817
	ClusterOne	0.961	0.552	0.670
	ProrankPlu	0.965	0.771	0.785
	Core&Peel	0.977	0.761	0.651
	IMHRC	0.963	0.590	0.691
	PC2P	0.949	0.582	0.646

	method	BP	MF	CC
Gavin	MCL	0.907	0.398	0.610
	MCODE	0.918	0.566	0.713
	CFinder	0.924	0.533	0.734
	AP	0.911	0.572	0.678
	CMC	0.957	0.558	0.726
	ClusterOne	0.969	0.558	0.713
	ProrankPlu	0.956	0.716	0.781
	Core&Peel	0.971	0.681	0.741
	IMHRC	0.973	0.579	0.699
	PC2P	0.959	0.594	0.771

	method	BP	MF	CC
KroganCore	MCL	0.768	0.354	0.405
	MCODE	0.896	0.519	0.597
	CFinder	0.930	0.487	0.617
	AP	0.846	0.542	0.594
	CMC	0.975	0.679	0.778
	ClusterOne	0.885	0.424	0.576
	ProrankPlu	0.927	0.628	0.701
	Core&Peel	0.880	0.696	0.749
	IMHRC	0.951	0.548	0.692
	PC2P	0.929	0.506	0.597

Supplementary Table 6. The average of enrichment score of predicted complexes with at least one enriched annotation over all clusters are compared for the nine approaches across all datasets.

	method	BP	MF	CC
KroganExt	MCL	0.810	0.441	0.499
	MCODE	0.894	0.723	0.660
	CFinder	0.901	0.405	0.570
	AP	0.879	0.637	0.621
	CMC	0.944	0.702	0.742
	ClusterOne	0.867	0.471	0.550
	ProrankPlu	0.897	0.551	0.627
	Core&Peel	0.918	0.709	0.775
	IMHRC	0.950	0.556	0.671
	PC2P	0.887	0.504	0.613

Human

	method	BP	MF	CC
STRING	MCL	0.868	0.424	0.679
	MCODE	0.884	0.465	0.736
	CFinder	0.911	0.563	0.830
	AP	0.827	0.383	0.678
	CMC	0.919	0.466	0.758
	ClusterOne	0.925	0.544	0.797
	ProrankPlu	0.935	0.576	0.809
	Core&Peel	0.949	0.474	0.682
	IMHRC	0.917	0.547	0.811
	PC2P	0.922	0.498	0.812

	method	BP	MF	CC
PIPS	MCL	0.846	0.500	0.526
	MCODE	0.895	0.561	0.737
	CFinder	0.942	0.609	0.783
	AP	0.912	0.573	0.754
	CMC	1.000	0.661	0.831
	ClusterOne	0.943	0.631	0.723
	ProrankPlu	0.967	0.640	0.804
	Core&Peel	1.000	0.724	0.846
	IMHRC	0.967	0.687	0.810
	PC2P	0.925	0.653	0.755

Supplementary Figure 1. Distribution of minimum and mean semantic similarities over clusters. The distribution of minimum and mean semantic similarities are given for (A – B) Gavin, (C – D) KroganCore, (E – F) KroganExt, (G - H) PIPS, as well as the mean for (I-J) Collin and STRING PPI networks.

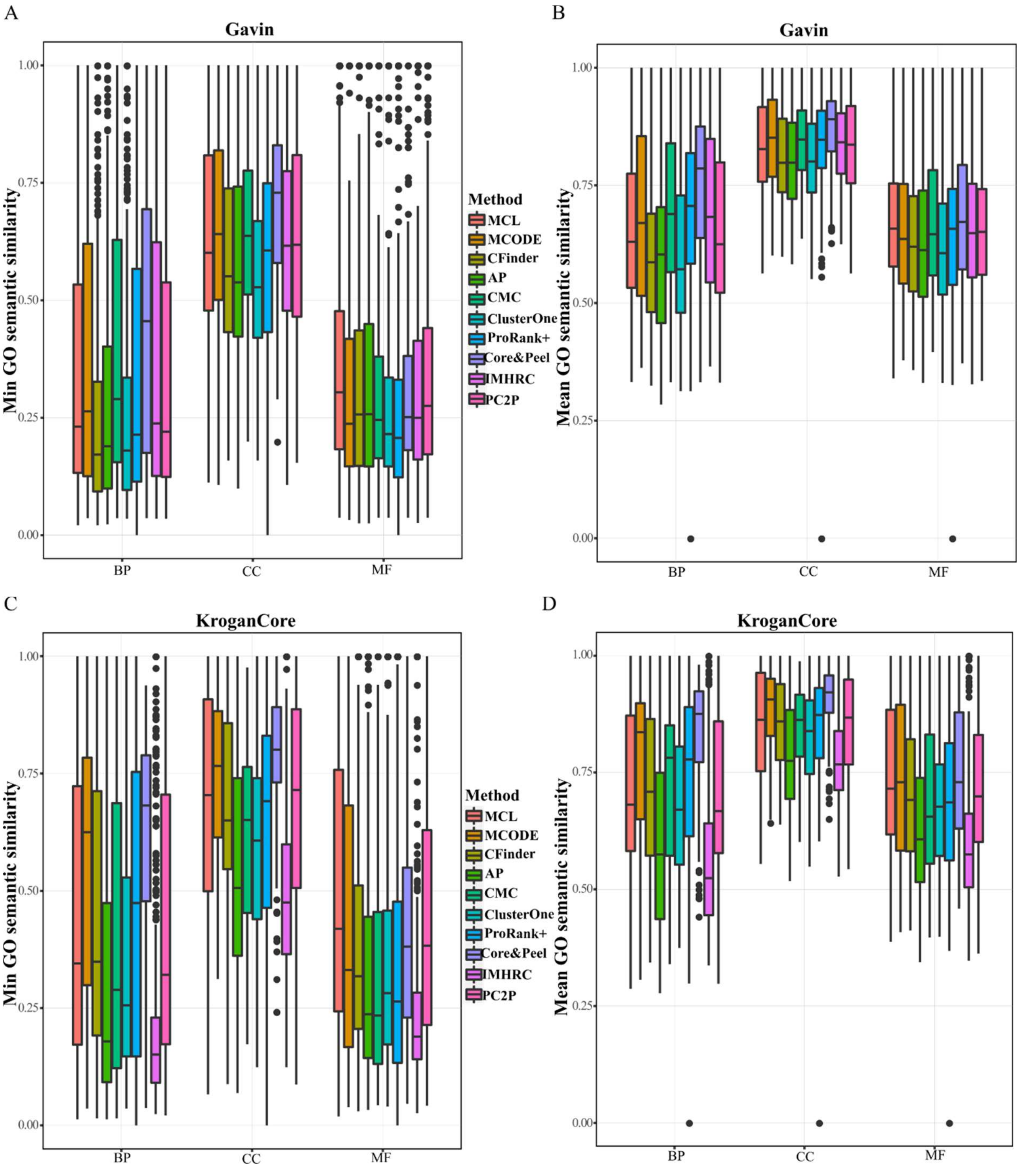

E

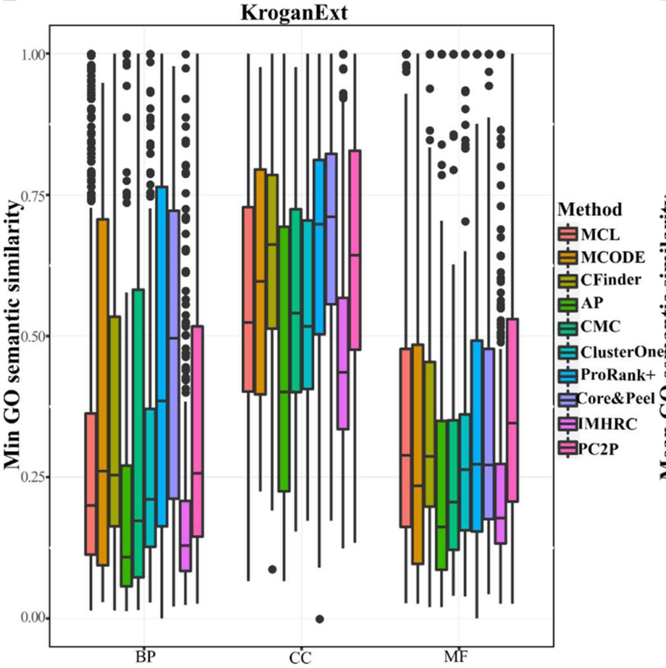
KroganExt
Min GO semantic similarity
0.00
0.25
0.50
0.75
1.00
BP
CC
MF
Method
MCL
MCODE
CFinder
AP
CMC
ClusterOne
ProRank+
Core&Peel
IMHRC
PC2P

F

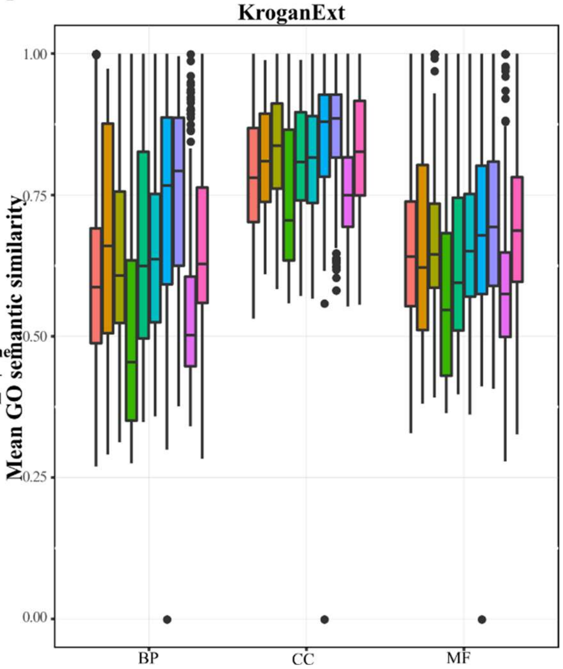
KroganExt
Mean GO semantic similarity
0.00
0.25
0.50
0.75
1.00
BP
CC
MF

G

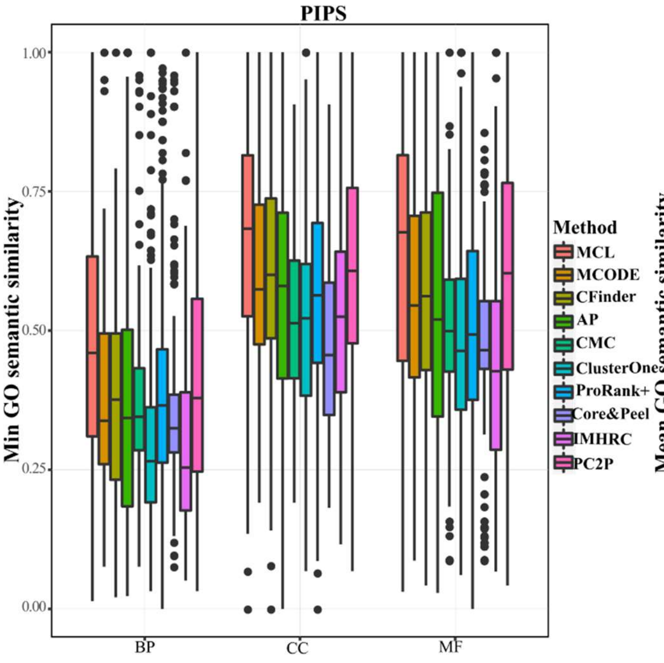
PIPS
Min GO semantic similarity
0.00
0.25
0.50
0.75
1.00
BP
CC
MF
Method
MCL
MCODE
CFinder
AP
CMC
ClusterOne
ProRank+
Core&Peel
IMHRC
PC2P

H

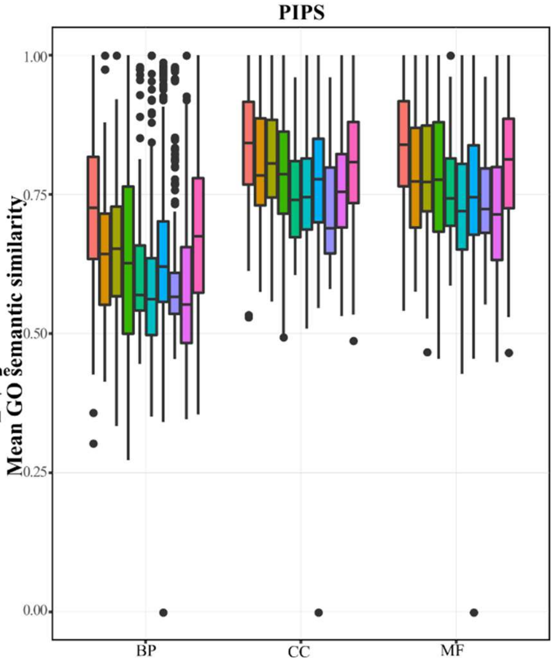
PIPS
Mean GO semantic similarity
0.00
0.25
0.50
0.75
1.00
BP
CC
MF

I

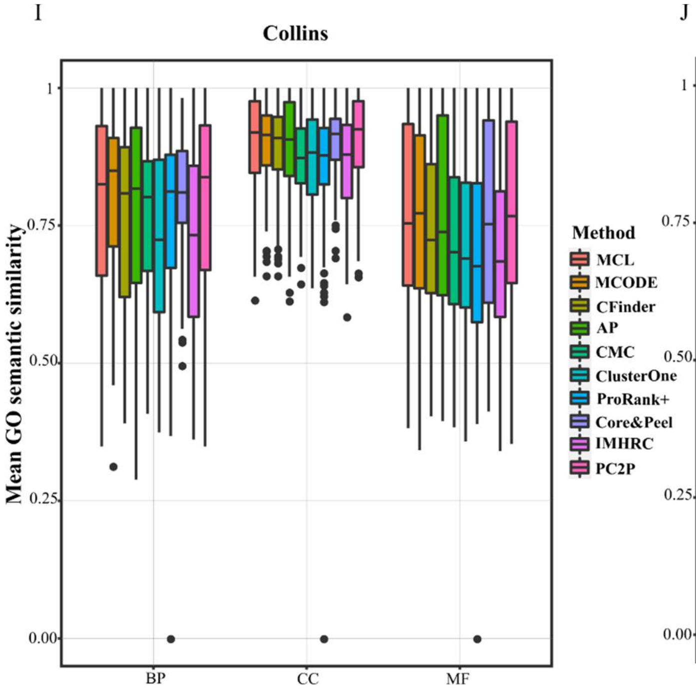
Collins
Mean GO semantic similarity
1
0.75
0.50
0.25
0.00
BP
CC
MF
Method
MCL
MCODE
CFinder
AP
CMC
ClusterOne
ProRank+
Core&Peel
IMHRC
PC2P

J

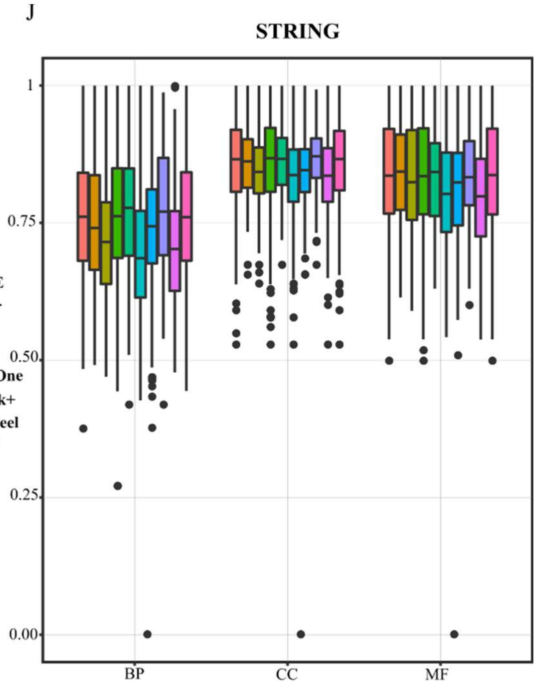
STRING
1
0.75
0.50
0.25
0.00
BP
CC
MF

Supplementary Figure 2. Comparative analysis of approaches for prediction of protein complexes in yeast. The comparative analyses is conducted with respect to a composite score combining five performance measures, maximum matching ratio (MMR), fraction match (FMR), separation (SEP), accuracy (ACC), and F-measure. Eight approaches, ordered by the year of publication, are compared on four PPI networks in yeast with respect to SGD gold standard. PCP2 outperforms all approaches on three of the networks.

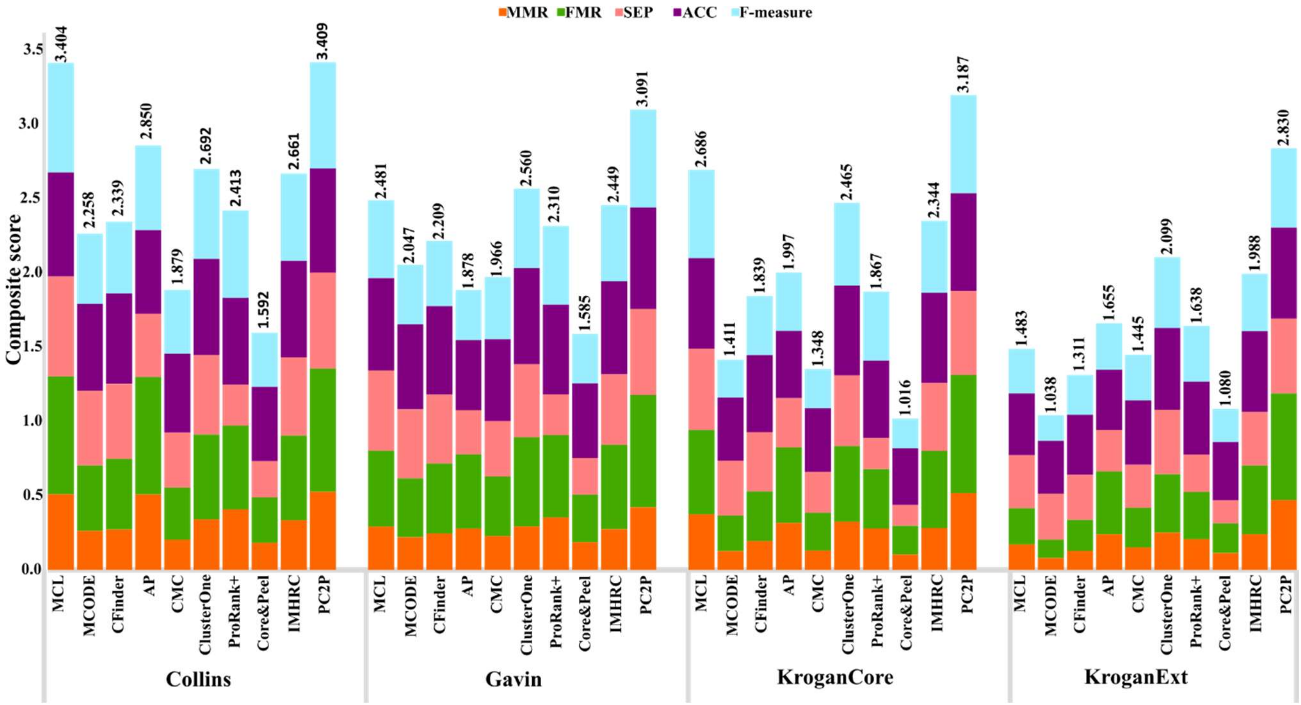

Supplementary Figure 3. Summation of MMr and F-measure+ of approaches for prediction of protein complexes. The sum of MMR and F-measure+ is conducted over the range of from 0 to 1. Ten approaches**,** ordered by the year of publication, are compared on (A-B) Collins, (C-D) KroganCore, (E-F) KroganExt, and (G-H) Gavin and PIPS in yeast and human respectively. PCP2 outperforms all approaches except in Collins.

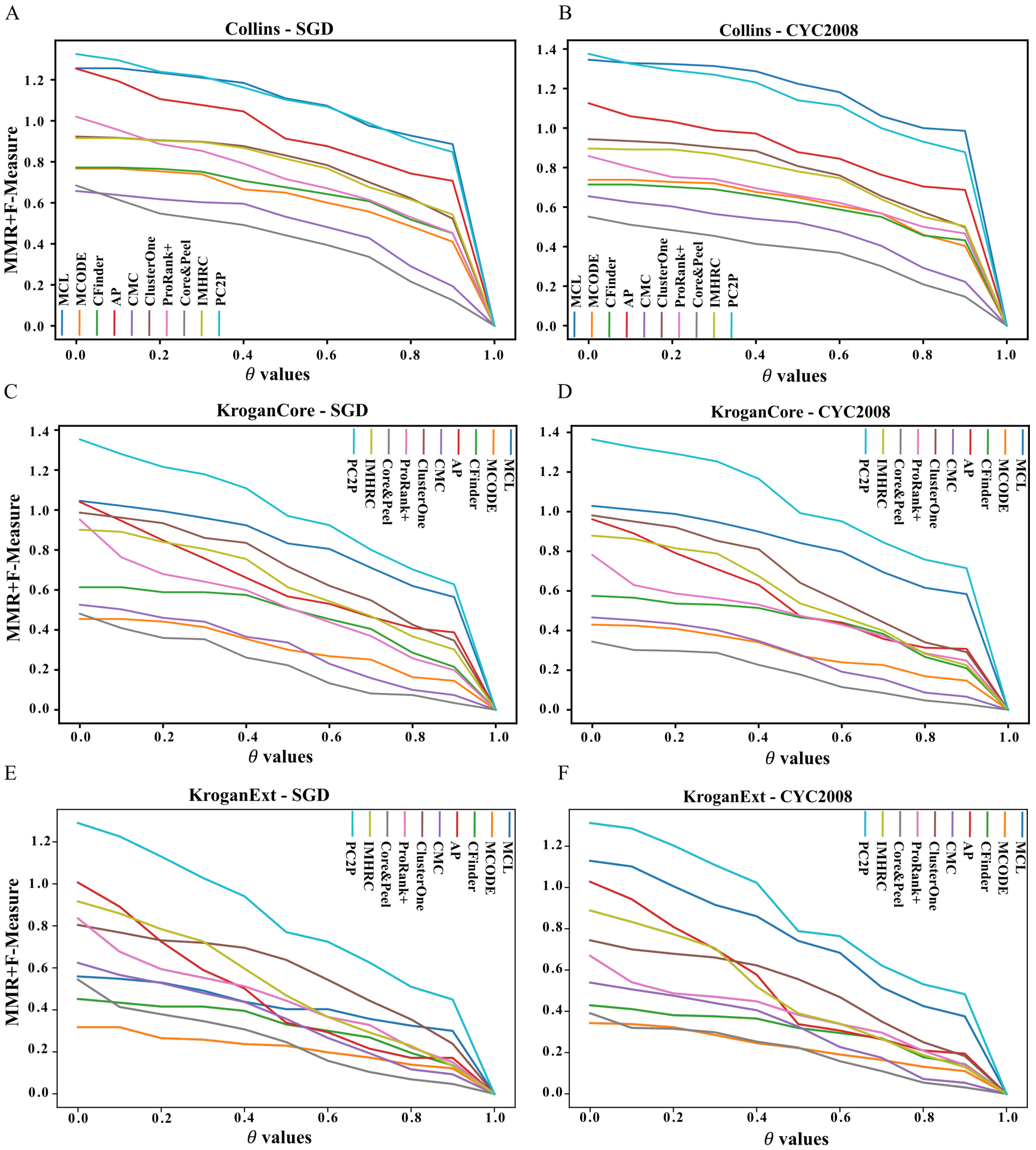

G

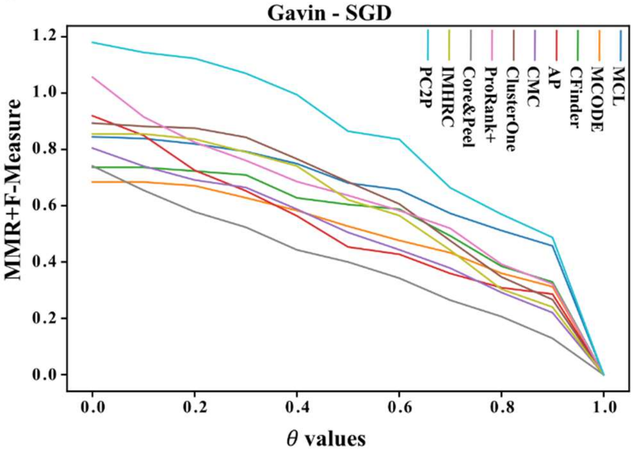
Gavin - SGD
MMIR+F-Measure
θ values
PC2P
IMHRC
Core&Peel
ProRank+
ClusterOne
CMC
AP
CFinder
MCODE
MCL

H

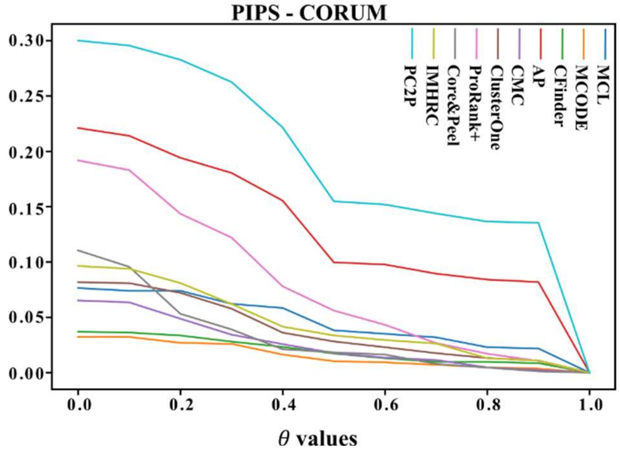
PIPS - CORUM
θ values
PC2P
IMHRC
Core&Peel
ProRank+
ClusterOne
CMC
AP
CFinder
MCODE
MCL

Supplementary Figure 4. Distribution of clusters size. The distribution of clusters size from ten approaches are compared on (A) Collins, (B) Gavin, (C) KroganCore, (D) KroganExt, (E) PIPS, and (F) STRING PPI networks. Distribution of cluster size of PC2P does not differ with respect to the compared approaches: For all algorithms, except ClusterOne, Core&Peel, and IMHRC, the cluster size distributions are monotonically decreasing by increasing the size of clusters.

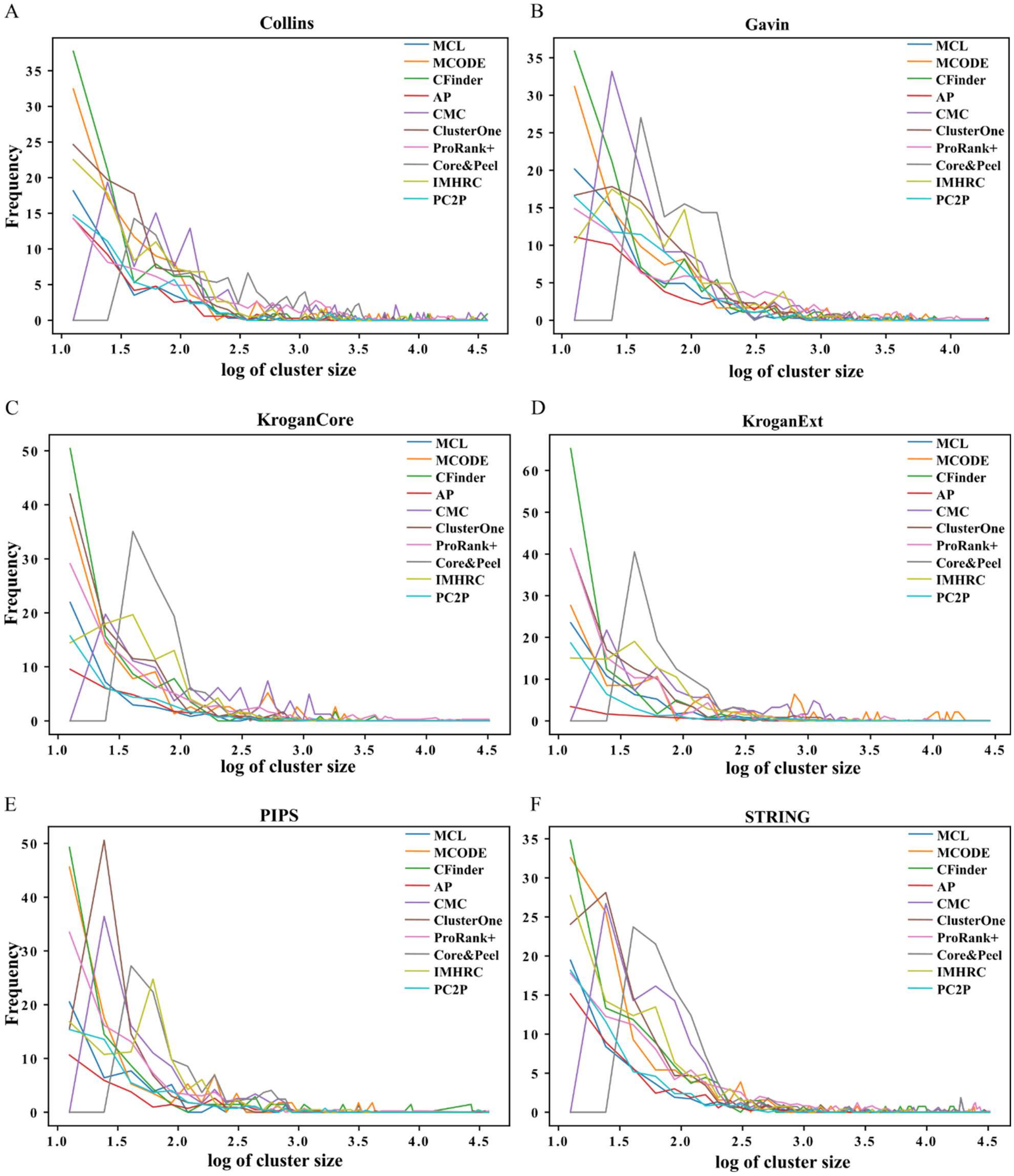

Supplement: btaa1089_Supplementary_Data [file btaa1089_supplementary_data.pdf]
